# Assessing the association between the Mediterranean, Dietary Approaches to Stop Hypertension and Mediterranean-DASH Intervention for Neurodegenerative Delay dietary patterns, structural connectivity and cognitive function

**DOI:** 10.1017/S0007114525000406

**Published:** 2025-04-14

**Authors:** Lizanne Arnoldy, Sarah Gauci, Lauren M. Young, Helen Macpherson, Oren Civier, Andrew Scholey, Andrew Pipingas, David J. White

**Affiliations:** 1 Centre for Mental Health and Brain Sciences, Swinburne University, Melbourne, Australia; 2 IMPACT – The Institute for Mental and Physical Health and Clinical Translation, Food & Mood Centre, School of Medicine, Deakin University, Geelong, Australia; 3 Centre of Research Excellence (CRE), Monash University, Melbourne, Australia; 4 Institute for Physical Activity and Nutrition (IPAN), School of Exercise and Nutrition Sciences, Deakin University, Geelong, VIC, Australia

**Keywords:** Ageing, Diet, Connectivity, Cognition, MRI

## Abstract

The rising incidence of neurodegenerative diseases in an ageing global population has shifted research focus towards modifiable risk factors, such as diet. Despite potential links between dietary patterns and brain health, inconsistencies in neuroimaging outcomes underscore a gap in understanding how diet impacts brain ageing. This study explores the relationship between three dietary patterns – Mediterranean, Dietary Approaches to Stop Hypertension (DASH) and Mediterranean-DASH Intervention for Neurodegenerative Delay – and cognitive outcomes as well as brain connectivity. The study aimed to assess the association of these diets with brain structure and cognitive function, involving a middle-aged healthy group and an older cohort with subjective cognitive decline. The study included cognitive assessments and diffusion-weighted MRI data to analyse white matter microstructural integrity. Participants comprised fifty-five older individuals with subjective cognitive decline (54·5 % female, mean age = 64) and fifty-two healthy middle-aged individuals (48·1 % female, mean age = 53). Age inversely correlated with certain cognitive functions and global brain metrics, across both cohorts. Adherence to the Mediterranean, DASH and Mediterranean-DASH Intervention for Neurodegenerative Delay diets showed no significant cognitive or global brain metric improvements after adjusting for covariates (age, education, BMI). Network-based statistics analysis revealed differences in brain subnetworks based on DASH diet adherence levels in the subjective cognitive decline cohort. In the healthy cohort, lower white matter connectivity was associated with reduced adherence to Mediterranean-DASH Intervention for Neurodegenerative Delay and DASH diets. Ultimately, the study found no strong evidence connecting dietary patterns to cognitive or brain connectivity outcomes. Future research should focus on longitudinal studies and refine dietary assessments.

As the global population ages, the prevalence of neurodegenerative conditions such as Alzheimer’s disease is escalating. With a lack of pharmaceutical solutions, researchers have focused on modifiable risk factors where accumulating evidence highlights the crucial role of diet in healthy cognitive and brain ageing^(1,2)^. Recent investigations, particularly those exploring the relationship between healthy dietary patterns that emphasise fruits, vegetables, legumes and nuts – such as the Mediterranean diet (MeDi)^(3)^, Dietary Approaches to Stop Hypertension (DASH)^(4)^ and Mediterranean-DASH Intervention for Neurodegenerative Delay (MIND) diet – have been positively associated with improved cognitive function, memory and executive function^(5–9)^. However, to this point, only a few studies have researched microstructural MRI measures of which only a limited number of studies have examined this association with the MeDi, which showed improved white matter connectivity and microstructure integrity across multiple tracts^(10)^. In contrast, the potential association between the DASH or MIND diets and white matter microstructure remains unexplored.

The MeDi consists primarily of olive oil, vegetables, fruits and legumes, with moderate consumption of fish and wine while limiting red meat and discretionary foods^(3)^. The DASH emphasises the high consumption of fruits, vegetables, grains, nuts and low-fat dairy products while limiting the consumption of sweets, saturated fatty acids, sugar-containing beverages and Na to reduce blood pressure^(4)^. The MIND diet combines components of the MeDi and DASH diets that have the most evidence for neuroprotection, promoting green leafy vegetables, other vegetables, berries, fish, poultry, beans, nuts, olive oil and wine while reducing red meats, fast and fried food, butter and margarine and cheese, as well as pastries and sweets^(5)^. The protective effect of these dietary patterns might be due to their emphasis on a high intake of antioxidant-rich foods such as vitamin C and resveratrol found in berries, grapes and nuts^(11)^. These antioxidants play a crucial role in lowering oxidative stress-induced damage and reducing amyloid-beta (A*β*) deposition^(12)^. Additionally, higher adherence to these dietary patterns is associated with reduced inflammation, metabolic abnormalities, insulin resistance and glucose levels, attributed to their high content of unsaturated fatty acids, fibre and essential nutrients^(13)^. These elements are important in vascular health, which is critical in maintaining optimal blood flow to the brain, thus potentially delaying or preventing the onset of cognitive decline.

A limited number of studies that have examined the association with the MeDi used older techniques of diffusion tensor imaging. Diffusion tensor imaging is a specific diffusion MRI method capable of detecting age-related changes in white matter microstructure, with studies reporting reduced fractional anisotropy and increased mean, radial and occasionally axial diffusivity in normal aging^(14–16)^ and subjective cognitive decline (SCD)^(17,18)^. To track the subtle, cumulative effects of diet on complex processes relevant to neurocognitive ageing, advanced neuroimaging measures are crucial for deepening our understanding of these relationships. Recently, there has been a noticeable trend towards incorporating the complete connectome into the analysis of brain networks, facilitating data-driven network identification aiming for a more comprehensive assessment of structural connectivity and improving our ability to detect early age-related changes^(19)^. While traditional network analysis methods like graph theory metrics have been underutilised, only one study utilised network-based statistic (NBS) analysis, which addresses issues of reduced statistical power from multiple comparison adjustments and enhances network specificity^(20,21)^. This study by Rodrigues *et al.* (2020) found heightened structural connectivity in networks involving the olfactory cortex, amygdala, calcarine, lingual and middle occipital gyri among individuals with high MeDi adherence^(22)^.

To better understand brain changes across the continuum of cognitive ageing, this study aims to investigate the role of the MeDi, DASH and MIND dietary patterns as a modifiable risk factor on white matter microstructural connectivity and cognitive function – by including a middle-aged healthy population and an at-risk older population with SCD. Specifically, the present study seeks to achieve four objectives: (1) assess the association between age, cognitive performance and brain connectivity, (2) examine the association between dietary patterns and cognitive performance measures, (3) investigate associations between topological measures using graph theory metrics resulting with dietary pattern adherence and (4) assess the presence of reduced subnetwork connectivity patterns via NBS and threshold-free network-based statistics (TFNBS) due to poor adherence to the MeDi, DASH or MIND diets.

## Materials and methods

### Study design

This present study integrated pre-intervention cross-sectional data from two separate randomised controlled trials to assess the above-mentioned association across the ageing continuum. Both trials were conducted by Swinburne University of Technology in Melbourne at the Centre of Human Pharmacology (now Centre for Mental Health and Brain Sciences). The first dataset, known as the Memory and Attention Supplement Trial (MAST), included a healthy middle-aged (HMA) population^(23)^. Additionally, the second trial, known as the Phospholipid Intervention for Cognitive Ageing Reversal (PLICAR) trial, included older at-risk individuals with SCD^(23,24)^. Each trial was centred around nutritional interventions in middle-aged and older individuals. The MAST trial investigated the effects of a 12-week-long trial of vitamin B and herbal supplements on cognition and mood, whereas the PLICAR trial examined the neurocognitive outcomes of a six-month supplementation using a phospholipid-rich milk protein (Lacprodan® PL-20, produced by Arla Foods Ingredients in Denmark). This study was conducted according to the guidelines laid down in the Declaration of Helsinki, and all procedures involving human patients were approved by the Swinburne University Human Research Ethics Committee (MAST: project number 2017–269; PLICAR: project number 2012-294). Written informed consent was obtained from all patients before enrolling in the clinical trial. Additionally, ethical approval from the Swinburne University Human Research Ethics Committee was received to utilise data from both trials for the present study (project number 20202924-4284). Both trials are registered with the Australian and New Zealand Clinical Trials Registry (PLICAR: ACTRN12613000347763) and (MAST: NCT03482063). The datasets were collected between May 2018 and September 2019 (MAST) and August 2014 and October 2017 (PLICAR).

### Participants

Eligibility criteria differed across the two trials. MAST included HMA participants free from suspected cognitive impairment, dementia and Alzheimer’s disease measured through a score above 24 on the Mini-Mental State Examination (MMSE) (hereafter referred to as the HMA cohort). The PLICAR trial included older individuals at risk for cognitive decline, adhering to age-associated memory impairment criteria. This included individuals experiencing SCD, determined by a Memory Complaint Questionnaire score ≥ 25^(25)^. Additionally, individuals included in the PLICAR trial were required to demonstrate a verbal memory performance score greater than one sd below the norm for healthy young adults^(24)^ (verbal paired associates test ≤ 32)^(26)^. A MMSE score above 24 was also required. These participants will henceforth be referred to as the SCD cohort. A detailed summary of the inclusion and exclusion criteria is provided in Table 1. Additionally, in the HMA cohort, a targeted advertising approach was implemented to diversify the dietary profiles of the recruited individuals. To achieve this, an assessment of participants’ diet quality was conducted using the Diet Screening Tool^(29)^ before enrolment, with assessments conducted up to 2 weeks prior to their baseline visit. Those scoring less than or equal to 59 were categorised as having a ‘suboptimal’ diet, while individuals scoring 60 or higher were classified as having an ‘optimal’ diet. In the MAST trial, half of the included participants in this trial were sought with an ‘optimal’ diet, while the other half had a ‘suboptimal’ diet^(23)^.

**Table 1.** Eligibility criteria of the SCD and HMA cohort

	SCD	HMA
Age	55–75	40–65
Cognitive function	Age-associated mild cognitive impairment	Free from any cognitive or neurological conditions
Inclusion	Fluent in English and free from a dementia diagnosis or any neurological, cardiac, endocrine, gastrointestinal or bleeding disorders	Free from neurological conditions or cognitive impairment (MMSE < 24), cardiac diseases, psychiatric disorders (including depression (BDI < 20) and anxiety), health conditions that could affect food absorption or events that could result in cognitive impairment (e.g. inflammatory bowel disease, coeliac disease, history of stroke, epilepsy, serious head trauma)
Exclusion	Smoker, a history of alcohol or substance abuse, use of cognitively affecting medications or substances, psychiatric illness, a rice allergy or a previous negative reaction to milk or dairy products	Smoker, colour blindness, uncontrolled hypertension, alcohol or substance abuse and the use of drugs, medication or supplements that could impact cognitive functioning
Dietary assessment tool	CCV FFQ	ASA24

SCD, subjective cognitive decline cohort; HMA, healthy middle-aged cohort; MMSE, Mini-Mental State Examination; BDI, Beck Depression Inventory; ASA24, Automated Self-Administered 24-Hour Dietary Assessment Tool^(27)^; CCV FFQ, Cancer Council Victoria FFQ^(28)^.

### Diet

The following section describes the dietary pattern scoring process (for more detail, see (30)).

#### Dietary assessment tool

The MAST and PLICAR trials used different dietary assessment methods to measure participants’ dietary intake.

In the HMA cohort, dietary data collection was facilitated through the Automated Self-Administered 24-Hour Dietary Assessment Tool (ASA24)^(31)^. In this tool, participants documented all the foods, drinks and dietary supplements that were consumed in the past 24 h. The assessment involved information about food form, preparation, portion size and any meal additions. Each reported item was assigned a specific food code. The foods and portion size options included in the ASA24 were guided by the Australian Food, Supplement and Nutrient Database (AUSNUT 2011–13), with data derived from the 2011–2013 Australian Health Survey. The AUSNUT 2011–12 covers a total of 5740 items. To deconstruct composite dishes into their constituent ingredients, the AUSNUT 2011–2013 recipe file was employed. This file provided information on the percentage of each ingredient present in various dishes and their total weights. The ingredients aligning with the MeDi, DASH and MIND dietary patterns were extracted from this disaggregated dataset.

Throughout the trial, participants completed the ASA24 on four occasions. Two assessments occurred before they received the investigative product, and two additional assessments took place during week 12 of the trial. These assessments spanned both weekdays and weekends, thereby accounting for potential variations in dietary habits across the week. All available recalls (ranging from a minimum of two to a maximum of four) were used to calculate the average intake and to assess adherence to the MeDi, DASH and MIND diets.

In the SCD population, participants’ regular dietary habits were evaluated using the Cancer Council Victoria FFQ (CCV FFQ), a validated tool for assessing individuals’ typical diets, which is validated in the Australian population^(27)^. Participants provided information on the frequency of consumption and portion sizes for a total of seventy-four food items and six types of beverages over the past 12 months^(32)^. The original data, including information on frequency and portion sizes, served as the basis for computing daily intake in grams. The calculation of nutrient and energy intake involved multiplying the consumption frequency of each item by its corresponding nutrient content, based on AUSNUT 2007 data.

#### Dietary patterns

Table 2 presents an overview of the MeDi, DASH and MIND dietary pattern scores. These scoring methodologies for each dietary pattern closely follow the scoring process outlined by Arnoldy *et al.* (2014), drawing from the original scoring methods by Martinez-Gonzales *et al.* (2012) for the MeDi, Folsom *et al.* (2017) for the DASH and Morris *et al.* (2015) for the MIND.

**Table 2.** Overview of the MeDi, DASH and MIND dietary pattern scores

	MeDi – Martinez-Gonzalez *et al.* (2012)			DASH – Folsom *et al.* (2007)			MIND – Morris *et al.* (2015)		
	Questions	Servings per d	Score	Component	Servings per d	Score	Components	Servings per d	Score
Olive oil and fats	Olive oil (%)	No	0	% kcal from fat	≥ 33	0	Olive oil*	Not primary oil	0
		Yes	1		> 30 to < 33	0·5		Primary oil	1
					≤ 30	1			
	Olive oil	< 13·5 g	0	% kcal from saturated fatty acids	≥ 13	0			
		≥ 13·5 g	1		> 10 to < 13	0·5			
					≤ 10	1			
Vegetables and fruits	Vegetables	< 2	0	Vegetable	< 2	0	Green leafy vegetables	≤ 0·29	0
		≥ 2	1		≥ 2 to < 4	0·5		> 0·29 to < 0·86	0·5
					≥ 4	1		≥ 0·86	1
	Fruit	< 3	0	Fruits	< 2	0	Other vegetables	< 0·71	0
		≥ 3	1		≥ 2 to < 4	0·5		≥ 0·71 to < 1	0·5
					≥ 4	1		≥ 1	1
							Berries	< 0·14	0
								≥ 0·14 to < 0·29	0·5
								≥ 0·29	1
Meat	Red meat, hamburger or meat products	> 1	0	Meats, poultry and fish	≥ 4	0	Red meat and products	≥ 1	0
		≤ 1	1		> 2 to < 4	0·5		≥ 0·57 to < 1	0·5
					≤ 2	1		< 0·57	1
	Chicken, turkey or rabbit meat (% of total meat intake)	≤ 50	0				Poultry	< 0·14	0
		> 50	1					≥ 0·14 to < 0·29	0·5
								≥ 0·29	1
	Fish or shellfish	< 0·43	0				Fish	< 0·033	0
		≥ 0·43	1					≥ 0·033 to < 0·14	0·5
								≥ 0·14	1
Dairy products	Butter, margarine or cream	> 1	0	Dairy products	< 1	0	Butter, margarine	> 2	0
		≤ 1	1		≥ 1 to < 2	0·5		≥ 1 to ≤ 2	0·5
					≥ 2	1		< 1	1
							Cheese	≥ 1	0
								≥ 0·14 to < 1	0·5
								< 0·14	1
Legumes and nuts	Legumes	< 0·43	0	Nuts, seeds and dry beans	< 0·29	0	Beans	< 0·14	0
		≥ 0·43	1		≥ 0·29 to < 0·57	0·5		≥ 0·14 to ≤ 0·43	0·5
					≥ 0·57	1		> 0·43	1
	Nuts	< 0·43	0				Nuts	< 0·033	0
		≥ 0·43	1					≥ 0·033 to < 0·71	0·5
								≥ 0·71	1
Grains				Total grains	< 5	0	Whole grains	< 1	0
					≥ 5 < 7	0·5		≥ 1 to < 3	0·5
					≥ 7	1		≥ 3	1
				Whole grains	< 1	0			
					≥ 1 to < 2	0·5			
					≥ 2	1			
Discretionary food and drinks	Sweet/carbonated beverages	≥ 1	0	Sweets	≥ 1·14	0	Fast/fried foods	≥ 0·57	0
		< 1	1		> 0·71 to < 1·14	0·5		≥ 0·14 to < 0·57	0·5
					≤ 0·71	1		< 0·14	1
	Commercial sweets or pastries (cakes, cookies, biscuits, custard)	≥ 0·43	0				Pastries, sweets	≥ 1	0
		< 0·43	1					≥ 0·71 < 1	0·5
								< 0·71	1
Alcohol	Wine	< 1	0				Wine	> 1 or 0	0
		≥ 1	1					> 0 to < 1	0·5
								1	1
Other	Vegetables, pasta, rice or other dishes seasoned with sofrito	< 0·29	0	Na	> 2401	0			
		≥ 0·29	1		> 1500 to ≤ 2401	0·5			
					≤ 1500	1			
Total score			14			11			15

MeDi, Mediterranean diet; DASH, Dietary Approaches to Stop Hypertension; MIND, Mediterranean-DASH Intervention for Neurodegenerative Delay.

Servings are presented in servings per d unless labelled differently. The serving sizes for each food item are presented in online Supplementary Tables 1–3 and are based on the original scoring methods of the diet. The serving sizes of items that were not reported in the original scoring method papers from the USDA National Nutrient Database for Standard Reference dietary guidelines (2015–2020) were utilised for food items and the National Institute on Alcohol Abuse and Alcoholism (NIAA) for alcoholic items (see Arnoldy *et al.* 2024 for a detailed description).

### Mediterranean diet

MeDi is a traditional dietary pattern emphasising olive oil as the primary fat source, along with vegetables, fruits, legumes, moderate fish and wine consumption and limited red meat and processed foods^(8)^. To measure adherence to MeDi, the study used the fourteen-item Mediterranean Diet Adherence Screener developed by Martinez-Gonzalez *et al.* (2012)^(3)^. It assigns a score of 1 or 0 to each of the fourteen dietary components and is known for its validation and applicability across diverse groups without population-specific cut-offs^(3,28)^. Adherence to MeDi was assessed based on the total score, which ranges from 0 to 14, with higher scores indicating stronger adherence^(3)^. Due to missing information about olive oil in the FFQ, the maximum score attainable for the CCV FFQ is 12 instead of 14. The identified food items included in each dietary assessment tool and the serving sizes utilised for each item (CCV FFQ and ASA24) are outlined in Table 1 of the online Supplementary Material.

### Dietary Approaches to Stop Hypertension

Adhering to the DASH diet is advised for reducing risk factors related to cardiovascular health, such as high blood pressure and elevated levels of lipid LDL-cholesterol, both of which are linked to an increased risk of dementia^(33,34)^. This dietary approach encourages a higher intake of fruits, vegetables, grains, nuts and low-fat dairy products, while concurrently discouraging the consumption of sweets, saturated fatty acids, sugary beverages and Na to manage high blood pressure^(4)^. To evaluate compliance with the low-Na DASH diet, we applied the DASH index developed by Folsom *et al.* (2007), including eleven components. The DASH score ranges from 0 to 11 with higher scores indicating high adherence. The identified food items included in each dietary assessment tool and the serving sizes utilised for each item (CCV FFQ and ASA24) are outlined in Table 2 of the online Supplementary material.

### Mediterranean-DASH Intervention for Neurodegenerative Delay

The MIND diet, developed by researchers at Rush University, draws inspiration from the MeDi and DASH diets while incorporating distinctive elements. The servings of fish and dairy products are adjusted and place a significant emphasis on the consumption of green leafy vegetables and berries to align with neuroprotective evidence^(5)^. To evaluate adherence to the MIND diet, we employed a combination of MIND scores constructed by Morris *et al.* (2015) and Meuller *et al.* (2020)^(35)^. The score ranged from 0 to 15 with higher scores indicating stronger adherence. Due to missing information about olive oil in the FFQ, the maximum score attainable for the CCV FFQ is 14 instead of 15. The MIND score presented in Table 2 has been slightly adapted from the original MIND score by Morris *et al.* (2015), presenting components in servings per d when possible. The identified food items included in each dietary assessment tool and the serving sizes utilised for each item (CCV FFQ and ASA24) are outlined in Table 3 of the online Supplementary material.

### Cognitive performance measures

#### Swinburne University Computerised Cognitive Ageing Battery

The Spatial Working Memory and Contextual Memory tasks from the Swinburne University Computerised Cognitive Ageing Battery were included due to the studied sensitivity in an ageing population^(36)^. A performance score was calculated by dividing the accuracy (%) by the response time (ms).

In the spatial working memory task, participants viewed a 4 × 4 white grid on a black background, with six white squares positioned in the grid for 3 s, followed by a blank grid. Four white squares were then shown sequentially in various positions of the grid. Participants indicated if they matched the original positions with a ‘yes’ or ‘no’ button. Participants completed fourteen trials each separated by a 2-s blank screen. Two out of four white squares match the original position in each trial. This task involved participants maintaining spatial information within their working memory.

In the contextual memory task, participants were presented with twenty images placed at different locations on the screen (top, bottom, left or right). Afterwards, the same images appeared in the centre of the screen, and participants had to identify their original positions using ‘top’, ‘bottom’, ‘left’ or ‘right’ button presses, which assessed participants’ episodic memory by having them recall the original spatial context.

#### Rapid visual information processing

In the rapid visual information processing (RVIP) task, participants are asked to continuously monitor a stream of single digits presented at a rate of 100 per min. They are tasked to identify a sequence of three consecutive odd or even digits and respond by pressing the ‘space bar’ as quickly as they can. Each run of the task lasted for 5 min, with eight correct targets per min. The RVIP was completed within the cognitive demand battery paradigm^(37)^, with three cycles completed over 30 min. RVIP performance was assessed by using the mean response time of correct detections and the mean of correctly detected target sequences.

### Imaging measures

#### MRI acquisitions

The MRI examination was performed at Swinburne University of Technology in Melbourne, Australia, utilising a Siemens 3 Tesla Tim Trio MRI scanner equipped with a 32-channel head coil. The anatomical scanning acquisitions were the same for both cohorts; however, the diffusion-weighted scans differed. The anatomical scans were obtained by using a T1-weighted structural image captured through a three-dimensional magnetisation-prepared rapid gradient echo sequence. The imaging parameters for the anatomical scans included a repetition time of 1900 ms, an echo time of 2·52 ms, isotropic resolution of 1 mm^3^ and a field of view of 256 × 256 mm.

For the HMA cohort, the diffusion-weighted scans were performed through two runs utilising a 2D echo-planar sequence using the following parameters: a repetition time of 8600 ms, echo time of 99 ms, field of view of 240 mm × 240 mm, 72 interleaved slices, multi-band factor of two and 2·0 mm³ isotropic voxels. For the SCD cohort, the diffusion-weighted scans were performed through two runs utilising a 2D echo-planar sequence with the following parameters: a repetition time of 9200 ms, echo time of 102 ms, field of view of 256 mm × 256 mm, 64 interleaved slices and isotropic voxels of 2·0 mm³. The initial acquisition involved sixty directions with a b-value of 2000 s/mm^2^, including ten b0 images. The second acquisition comprised thirty directions at a b-value of 900 s/mm². Prior to pre-processing, the neuroimaging data were reorganised to conform to the Brain Imaging Data Structure^(38)^.

#### MRI data pre-processing and connectome construction

**Figure 1.**
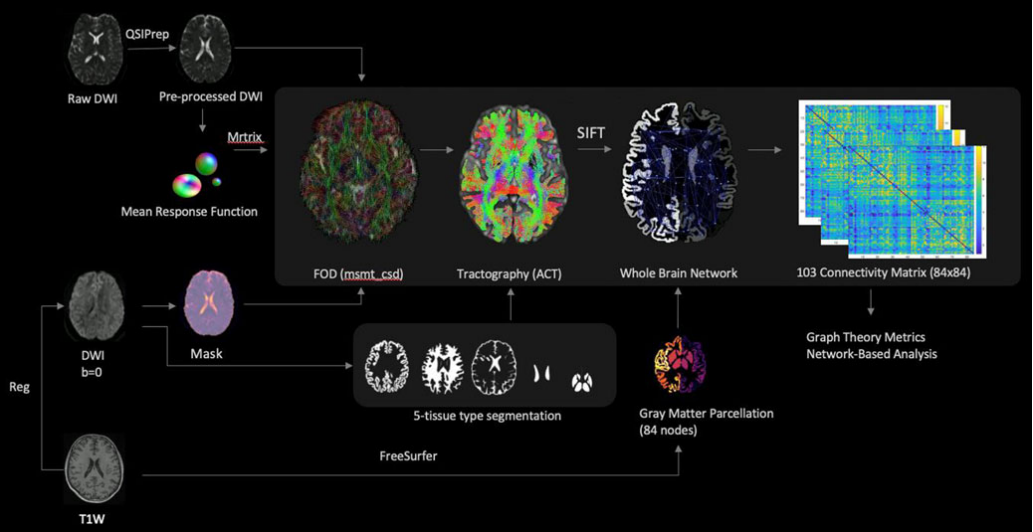
This figure illustrates the processing pipeline used in this study. Initially, the T1-weighted (T1W) and diffusion-weighted images (DWI) underwent pre-processing using the Quantitative Susceptibility Imaging Preparation (QSIPrep) pipeline and FreeSurfer. Subsequently, the QSIPrep reconstruction pipeline was utilised to obtain the response functions. The mean response function was then computed, followed by estimating the fibre orientation distributions (FOD). To generate a whole-brain tractography, the five-tissue type segmentation and normalised FOD were incorporated. Additionally, anatomically constrained tractography (ACT) was applied to enhance the biological plausibility. To reduce the number of streamlines, spherical-deconvolution-informed filtering of tractograms 2 (SIFT2) was employed. Finally, for each participant in both the healthy and diabetic datasets, symmetric N × N undirected weighted connectivity matrices were constructed. These matrices were based on the Desikan-Killiany atlas, consisting of eighty-four cortical and subcortical regions (nodes). Network-based statistics and graph theory metrics were then computed and compared between the groups in a cross-sectional analysis (copyright from include citation: Arnoldy *et al.*).

The dataset underwent pre-processing with Quantitative Susceptibility Imaging Preparation (QSIPrep)^(39)^ according to best practices in the field, with an extensive description available in online Supplementary Material Section C. Connectomes for each dataset were consistently generated using QSIPrep 0·16·1^(39)^ and MRtrix3 (v3·0·3_20210917)^(40)^, with the processes run on the Neurodesk platform^(41)^. The workflow entailed computing the mean response function and implementing multi-shell, multi-tissue constrained spherical deconvolution using the Dhollander algorithm. Normalisation of fibre orientation distributions was achieved via mtnormalize, and streamlines were generated through the iFOD2 algorithm, paired with anatomically constrained tractography and the spherical-deconvolution informed filtering of tractograms 2 (SIFT2) method for tractogram filtering, from which the SIFT proportionality coefficient (mu-value) was derived. An 84 × 84 connectivity matrix for each individual was constructed using eighty-four anatomical regions of interest, based on FreeSurfer-derived parcellations^(42)^. A visual summary of the connectivity matrix construction is presented in Fig. 1.

### Measures

#### Global metrics

In the present study, graph theory metrics in their weighted undirected form were investigated. These metrics included global efficiency, average local efficiency, modularity and assortativity computed using the Brain Connectivity Toolbox (v03/03/2019)^(43)^. Additionally, metrics for clustering coefficient, characteristic path length, normalised clustering coefficient, normalised characteristic path length, small-worldness and vulnerability were determined using the methods established by Yeh *et al.* (2016), as they demonstrated their suitability for dense weighted connectomes^(44)^. All graph theory metrics were created through MATLAB R2022b^(45)^, and the SIFT proportionality coefficient (mu-value) was obtained during the reconstruction workflow.

#### Network-based statistics

The NBS method was applied to assess differences in individuals’ connectivity matrices across dietary adherence groups by comparing average streamline counts between the lowest tertile and higher adherence groups (middle and high) for the MeDi, DASH and MIND diets. NBS is a nonparametric statistical technique developed to control family-wise error rates^(21)^. A detailed explanation of the NBS method can be found in the study by Zalesky *et al.* (2010). Besides NBS, this paper utilised TFNBS^(46)^. TFNBS is an improved method that incorporates threshold-free cluster enhancement to reduce threshold dependence, emphasising interconnected edges over spatial clusters. A comprehensive description can be found in the study by Baggio *et al.* ^(46)^.

### Statistical analysis

Statistical analyses were conducted using MRtrix3 for NBS and TFNBS, and IBM SPSS Version 29·0 was employed for the analyses including cognitive outcomes and global brain metrics. Continuous variables representing demographic and sample characteristics are presented through means and sd, whereas percentages were used to report categorical variables. To identify potential outliers, values significantly deviating from the overall data distribution were recognised and treated as missing values (*n* 2 in both datasets). Outliers in both mu-value and streamline count within the connectome data were visually identified via scatterplots, and participants exceeding 3 sd from the mean were excluded, and Mahalanobis distance outliers were checked.

Power analysis for our primary hierarchical regression indicated adequate power (0·80) to detect large and medium effects with our sample size of fifty-five and fifty-two participants. However, research has demonstrated that analysing individual graph metrics in case–control studies requires a minimum of sixty-five subjects per group to obtain sufficient statistical power of 80 %^(47)^. To address the challenges of multiple comparisons in network analyses, we employed NBS and TFNBS, which help maintain statistical power while improving network detection precision. Additionally, we conducted extensive robustness testing across multiple thresholds and graph densities.

The current statistical analysis aimed to investigate the association between various healthy dietary patterns (MeDi, DASH and MIND), cognitive outcomes and white matter integrity in two separate datasets. The first dataset included individuals with SCD (aged 56–75) and the second dataset included HMA individuals (aged 40–65). Initially, correlations using Pearson’s method were explored between the continuous dietary pattern scores, cognitive measures and global brain metrics. Additionally, hierarchical regression analyses were carried out, where age and dietary scores served as the independent variable, and cognitive outcomes and global metrics were entered as the dependent variable while adjusting for covariates. The covariates considered in the analysis were age (in years), years of education and BMI. All were selected due to their known associations with cognition and cognitive decline^(48–50)^. A significance level *of P* < 0·05 was utilised, and false discovery rate correction (q = 0·05) was employed to address multiple comparisons, following the method proposed by Benjamini and Hochberg in 1995^(51)^.

To evaluate structural connectivity differences among adherence groups for the MeDi, DASH and MIND dietary patterns in both datasets, NBS and TFNBS analyses using the MRtrix3 connectomestats command were employed. NBS and TFNBS analyses used data-driven dietary pattern adherence groups to contrast low-, middle- and high-adherence tertile groups. The analysis incorporated a model including group assignment (low, middle and high adherence), and the covariates (age, years of education and BMI) were conducted. A two-sample *t* test was performed on each edge, comparing average streamline counts between individuals in the lowest tertile group compared with those in the highest or middle adherence group. In line with the shown significant results by Rodrigues *et al.*’s (2020) paper, the present paper computed the primary analysis with the following settings: 30 % density, *t*-threshold: 3·5 (e.g. *P* = 0·0005, comparable to a *t* = 3·5, one-tailed hypothesis test). Follow-up analyses were run with the following settings: 80 and 95–100 % density and *t*-thresholds of 2·38 and 3 (comparable to the *P* = 0·01 and *P* = 0·001 in the paper by Rodrigues *et al.* (2020)). For TFNBS, the initial analysis was run with the following settings: 30 % density, and the enhancement parameters E and H were set at 0·75 and 3. The follow-up analysis adjusted the density mask to 80 and 95–100 %. Both NBS and TFNBS determined significant connected components through 5000 permutations, reported at a family-wise error-corrected *P*-value of 0·05.

## Results

### Characteristics of the included cohorts

Table 3 provides an overview of the demographic characteristics of both datasets. The SCD dataset included thirty female participants (54·5 %) and twenty-five male participants (45·5 %), with a mean age of 64 (sd ± 4·73) years. Most participants demonstrated low-to-moderate adherence to the respective dietary patterns, indicated by an average score of 3·5 out of 12 for the MeDi, 3·72 out of 11 for the DASH diet and 7·65 out of 14 for the MIND diet. The HMA cohort comprised twenty-five female participants (48·1 %) and twenty-seven male participants (51·9 %), with an average age of 53 (±6·45) years. The majority of participants in the HMA cohort exhibited low-to-moderate adherence to the specific dietary patterns, as reflected by an average score of 5·08 out of 14 for the MeDi, 4·71 out of 11 for the DASH diet and 6·84 out of 15 for the MIND diet. The details on the ranges and participant distribution for the data-driven dietary pattern groups utilised for the NBS and TFNBS analysis are provided in online Supplementary material D Table S4.

**Table 3.** Characterisation of the SCD and HMA cohort

	SCD					HMA				
	*n*	Minimum	Maximum	Mean	sd	*n*	Minimum	Maximum	Mean	sd
Sex (% female)	55			54·50		52			48·10	
Age	55	56·5	74·8	63·89	4·7	52	40·3	64·9	53·1	6·5
Education (years)	55	9·0	21·0	15·6	3·0	52	12·0	23·0	17·0	2·9
CVD measures										
BMI	55	20·2	44·1	28·7	4·5	52	19·6	40·6	27·8	5·1
Diet										
Energy (KJ)	52	3953	14 163	7051	2164	52	5084	18 726	9505	2603
MeDi dietary pattern	52	1·00	6·00	3·50	1·32	52	2·00	9·00	5·08	1·61
DASH dietary pattern	52	0·00	7·50	3·72	1·38	52	1·50	7·00	4·71	1·49
MIND dietary pattern	52	5·00	10·00	7·65	1·29	52	3·00	11·50	6·84	1·99

SCD, subjective cognitive decline cohort; HMA, healthy middle-aged cohort; *n*, number of participants; MeDi, Mediterranean diet; DASH, Dietary Approaches to Stop Hypertension; MIND, Mediterranean-DASH Intervention for Neurodegenerative Delay.

Demographic characteristics of both datasets.

### Healthy middle-aged cohort

#### Correlations

In the HMA cohort, age exhibited a negative association with contextual memory (Table 4). Additionally, positive associations were identified among the scores of each dietary pattern. No significant correlations were observed between the MeDi, DASH and MIND and global metrics or cognitive outcomes.

**Table 4.** Correlations between dietary patterns, graph theory metrics and cognitive measures in the MAST dataset

	MeDi	DASH	MIND	Mu value	Modularity	Assortativity	Small worldness	Gamma	Lambda	Global efficiency	Mean local efficiency	Mean CC	Mean path length	Mean vulnerability	MMSE	Spatial working memory	Contextual memory	RVIP Acc	RVIP TR
Age	−0·16	0·08	−0·17	0·05	0·08	−0·02	0·07	0·16	0·12	−0·14	−0·15	−0·07	0·23	−0·04	−0·13	−0·05	−0·423**	−0·052	0·08
MeDi		0·433**	0·588**	−0·08	0·08	0·09	0·12	−0·03	−0·15	0·19	0·09	0·03	−0·11	0·14	−0·245	−0·03	−0·03	−0·05	−0·09
DASH			0·508**	−0·07	−0·15	−0·06	0·18	0·04	−0·03	0·05	0·06	0·12	−0·02	0·01	−0·137	−0·06	−0·21	−0·03	−0·14
MIND				−0·14	0·00	0·02	−0·05	0·07	0·12	0·10	0·11	−0·01	0·14	0·00	−0·202	−0·2	−0·22	0·16	−0·14
Mu value					0·076	−0·114	0·269	0·145	0·017	−0·602**	−0·373**	−0·123	0·313*	−0·067	0·01	0·456**	0·18	0·07	0·19
Modularity						−0·02	0·031	0·045	0·082	−0·207	−0·565**	−0·089	0·222	0·305*	0·01	0·06	−0·13	0·05	0·09
Assortativity							−0·022	−0·566**	−0·544**	0·104	0·268	0·182	0·018	0·346*	−0·21	−0·25	−0·11	−0·20	−0·03
Small worldness								0·287*	−0·383**	−0·188	−0·174	−0·05	−0·318*	−0·064	−0·04	0·19	−0·02	−0·08	−0·03
Gamma									0·722**	−0·076	−0·350*	0·111	0·1	−0·213	0·06	0·24	0·00	0·15	0·09
Lambda										0·042	−0·262	0·15	0·291*	−0·123	0·04	0·13	−0·04	0·19	0·15
Global efficiency											0·491**	0·298*	−0·572**	0·259	−0·05	−0·23	0·03	−0·04	−0·19
Mean local efficiency												−0·066	−0·372**	−0·235	−0·09	−0·276*	0·01	−0·08	−0·20
Mean CC													0·061	0·313*	0·13	−0·10	0·06	0·00	0·07
Mean path length														−0·008	−0·05	−0·02	−0·18	−0·03	0·14
Mean vulnerability															−0·01	0·17	−0·02	−0·04	−0·15

MeDi, Mediterranean diet; DASH, Dietary Approaches to Stop Hypertension; MIND, Mediterranean-DASH Intervention for Neurodegenerative Delay; CC, clustering coefficient; MMSE, Mini-Mental State Examination; RVIP, rapid visual information processing; Acc, accuracy; BL, Bond-Lader; TR, response time.

Spearman’s correlation coefficient, *P < 0·05, **P < 0·01.

The mu-value demonstrated positive associations with special working memory, and mean local efficiency was negatively associated with spatial working memory.

#### Relationship between age and cognitive outcomes and global metrics

To examine the association between age, cognitive outcomes and global metrics, a hierarchical regression analysis was performed controlling for years of education and BMI (Tables 5 and 6). The regression coefficient and standard error for each analysis are shown. Increased age was associated with reduced spatial working memory (*P* = 0·009) and contextual memory (*P* = 0·023) (Table 5). Age was not associated with global brain metrics (Table 6).

**Table 5.** Association of age with cognitive scores, standardised coefficients beta and adjusted R-square

	MMSE		Spatial working memory		Contextual memory		RVIP Acc		RVIP TR	
	*β*	R^2^	*β*	R^2^	*β*	R^2^	*β*	R^2^	*β*	R^2^
Age	0·019	–0·029	–0·371**	0·129	–0·344*	0·053	–0·224	0·007	0·068	–0·026

MMSE, Mini-Mental State Examination; RVIP, rapid visual information processing; Acc, accuracy; TR, response time.

Hierarchical regressions controlling for years of education and BMI. None yielded significant results after false discovery rate correction. *P < 0·05, **P < 0·01.

**Table 6.** Association of age with graph theory metrics, standardised coefficients beta and adjusted R-square

	Mu value		Modularity		Assortativity		Small worldness		Normalised CC		Normalised path length		Global efficiency		Mean local efficiency		Mean CC		Mean path length		Mean vulnerability	
	*β*	R^2^	*β*	R^2^	*β*	R^2^	*β*	R^2^	*β*	R^2^	*β*	R^2^	*β*	R^2^	*β*	R^2^	*β*	R^2^	*β*	R^2^	*β*	R^2^
Age	0·061	0·066	0·032	–0·024	0·012	–0·056	0·142	0·046	0·205	0·064	0·025	–0·054	–0·147	0·005	–0·106	0·047	–0·069	–0·041	0·145	–0·037	0·02	–0·03

CC, clustering coefficient.

Hierarchical regressions controlling for years of education and BMI.

#### Relationship between dietary pattern scores and cognitive scores

The association between dietary patterns and cognitive outcomes was examined using hierarchical regressions while accounting for age, years of education and BMI as control variables (refer to Table 7). The analysis did not reveal any significant relationships between dietary patterns and MMSE, spatial working memory, contextual memory or RVIP.

**Table 7.** Association of dietary pattern scores with cognitive scores, standardised coefficients beta and adjusted R-square

	MMSE		Spatial working memory		Contextual memory		RVIP Acc		RVIP TR	
	*β*	R^2^	*β*	R^2^	*β*	R^2^	*β*	R^2^	*β*	R^2^
MeDi	–0·277^	0·028	0	0·111	–0·086	0·04	0·001	–0·015	–0·12	–0·034
DASH	–0·152	–0·027	0·023	0·111	–0·126	0·048	0·034	–0·014	–0·284^	0·033
MIND	–0·219	0	–0·192	0·15	–0·182	0·068	0·156	0·01	–0·172	–0·017

MMSE, Mini-Mental State Examination; RVIP, rapid visual information processing; Acc, accuracy; TR, response time; MeDi, Mediterranean diet; DASH, Dietary Approaches to Stop Hypertension; MIND, Mediterranean-DASH Intervention for Neurodegenerative Delay.

Hierarchical regressions controlling for age, years of education and BMI. ^*P* < 0·10.

#### Relationship between dietary patterns and global brain metrics

The hierarchical regressions conducted to assess the relationship between dietary patterns and global brain metrics did not unveil any significant associations (see Table 8).

**Table 8.** Association of dietary pattern scores with graph theory metrics, standardised coefficients beta and adjusted R-square

	Mu value		Modularity		Assortativity		Small worldness		Normalised CC		Normalised path length		Global efficiency		Mean local efficiency		Mean CC		Mean path length		Mean vulnerability	
	*β*	R^2^	*β*	R^2^	*β*	R^2^	*β*	R^2^	*β*	R^2^	*β*	R^2^	*β*	R^2^	*β*	R^2^	*β*	R^2^	*β*	R^2^	*β*	R^2^
MeDi	−0·098	0·056	−0·003	−0·046	0·083	−0·071	0·205	0·069	0·025	0·044	−0·155	−0·052	0·131	0·001	0·075	0·033	−0·064	−0·059	−0·12	−0·044	0·128	−0·036
DASH	−0·111	0·059	−0·172	−0·016	−0·074	−0·073	0·063	0·029	0·001	0·044	−0·059	−0·073	0·16	0·009	0·157	0·052	−0·064	−0·059	−0·122	−0·044	−0·002	−0·052
MIND	−0·102	0·057	−0·079	−0·039	0·036	−0·077	0·025	0·026	0·149	0·067	0·084	−0·069	0·083	−0·009	0·073	0·033	−0·079	−0·057	0·111	−0·046	0·006	−0·052

CC, clustering coefficient; MeDi, Mediterranean diet; DASH, Dietary Approaches to Stop Hypertension; MIND, Mediterranean-DASH Intervention for Neurodegenerative Delay.

Hierarchical regressions controlling for age, years of education and BMI. None yielded significant results before or after false discovery rate correction.

#### Relationship between dietary pattern scores and network-based statistics/threshold-free network-based statistics outcomes

**Figure 2.**
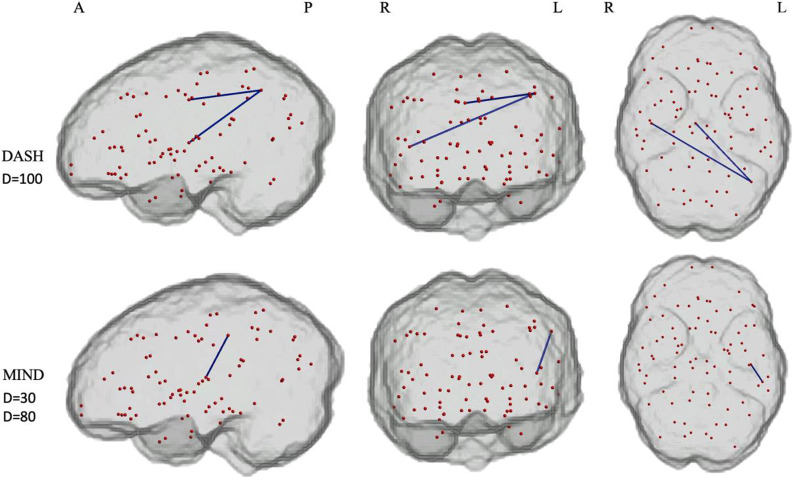
Subnetworks with reduced connectivity in individuals adhering to the lowest tertile of the DASH and MIND dietary pattern compared with individuals in the middle tertile group in the HMA cohort. Weakened connections are highlighted in blue edges, and nodes are presented in red, which are all equal-sized. This analysis is based on the sensitivity analysis with a 100 % density mask in the analysis assessing the DASH and a 30 and 80 % density mask in the analyses assessing the MIND and presents the results of the analysis with a threshold of 3·5 (controlled for covariates age, BMI and year of education). DASH, Dietary Approaches to Stop Hypertension; MIND, Mediterranean-DASH Intervention for Neurodegenerative Delay; HMA, healthy middle-aged. A, anterior; L, left hemisphere; P, posterior; R, right hemisphere.

In the initial NBS analysis in the HMA cohort, employing a threshold of 3·5 and a density mask of 30, the MIND diet showed significantly lower connectivity between the left supramarginal gyrus and left transverse temporal gyrus in the lowest tertile compared with the middle tertile group (Fig. 2 and Table 9). No additional, significant differences in brain connectivity were found when comparing low-adherence groups to middle or high-adherence groups for the MeDi and DASH dietary patterns.

**Table 9.** Connectivity differences between low adherence and middle tertile adherence in DASH and MIND patterns

Dietary pattern	Density mask	Nodes		t	*P*
DASH	100	7 – 71	Left inferior parietal to right posterior cingulate	5·07	0·03
		7 – 82	Left inferior parietal to right transverse temporal	4·07	0·03
MIND	30	30 – 33	Left supramarginal to left transverse temporal	4·51	0·012
	80	30 – 33	Left supramarginal to left transverse temporal	4·51	0·043

DASH, Dietary Approaches to Stop Hypertension; MIND, Mediterranean-DASH Intervention for Neurodegenerative Delay.

This table displays the *t*-value and corresponding *P*-values from the network-based statistics analysis. The analysis applied a statistical significance level of *P* = 0·05 (threshold = 3·5, one-tailed *t* test) to identify edges exceeding the threshold within interconnected components. The statistical significance of each detected component was assessed using an empirical null distribution derived through 5000 permutations.

In the subsequent sensitivity analysis, increasing the density mask to 80 % upheld the reduced connectivity finding for the MIND diet. However, after increasing the density to the original 95–100 %, the difference was no longer observed. At this original density, two connections showed significantly lower connectivity in the low-adherence group of the DASH diet compared with the middle tertile. These connections were identified between nodes in the left inferior parietal and right posterior cingulate, as well as between the left inferior parietal and right transverse temporal (Fig. 2 and Table 9). After lowering the threshold no additional significant differences were found.

The TFNBS analysis found no significant connectivity differences.

### Subjective cognitive decline cohort

#### Correlations between variables

The preliminary analysis explored the correlation between each dietary pattern, global metrics and cognitive scores. The results of Spearman’s correlations are elaborated in Table 10 for the SCD cohort. Age exhibited a negative association with the mu-value and contextual memory while showing a positive association with mean vulnerability. Additionally, positive associations were identified among the scores of each dietary pattern. Adherence to the MeDi was found to be negatively associated with assortativity. No significant correlations were observed between DASH and MIND and global metrics or cognitive outcomes.

**Table 10.** Correlations between dietary patterns, graph theory metrics and cognitive measures in the PLICAR dataset

	MeDi	DASH	MIND	Mu value	Modularity	Assortativity	Small worldness	Gamma	Lambda	Global efficiency	Mean local efficiency	Mean CC	Mean path length	Mean vulnerability	MMSE	Spatial working memory	Contextual memory	RVIP Acc	RVIP TR
Age	–0·161	0·077	–0·167	–0·335*	–0·168	0·153	0·128	–0·03	–0·164	0·053	0·083	–0·122	–0·002	0·275*	–0·13	–0·052	–0·423**	–0·052	0·076
MeDi		0·23	0·32*	0·16	0·17	–0·293*	–0·05	0·07	0·18	–0·09	–0·21	0·23	0·26	0·03	–0·09	0·07	0·10	0·05	–0·16
DASH			0·44**	–0·03	0·02	–0·12	0·21	0·20	0·12	–0·13	–0·21	–0·03	0·16	0·10	–0·27	–0·15	–0·07	–0·04	0·03
MIND				0·14	0·13	–0·13	0·12	–0·03	–0·07	–0·11	–0·10	0·06	0·12	0·04	–0·18	–0·23	0·26	–0·18	–0·09
Mu value					0·500**	–0·208	–0·068	–0·035	0·084	–0·343*	–0·461**	0·249	0·271*	–0·209	0·15	0·19	0·11	0·14	–0·277*
Modularity						–0·384**	0·234	0·280*	0·229	–0·206	–0·593**	0·142	0·116	0·185	0·10	–0·10	0·12	–0·26	0·01
Assortativity							–0·319*	–0·459**	–0·392**	0·014	0·376**	–0·091	–0·067	–0·046	0·01	0·04	0·05	–0·12	0·25
Small worldness								0·671**	0·049	–0·083	–0·280*	–0·143	–0·158	0·209	–0·10	–0·10	–0·08	0·03	0·08
Gamma									0·718**	–0·113	–0·441**	–0·142	0·089	0·11	–0·11	0·06	–0·07	0·03	0·06
Lambda										–0·054	–0·369**	0·009	0·245	0·018	0·01	0·13	0·04	–0·04	0·06
Global efficiency											0·528**	0·383**	–0·783**	0·062	–0·01	–0·10	–0·15	–0·07	0·18
Mean local Efficiency												0·022	–0·404**	–0·343*	0·16	–0·13	–0·13	0·11	0·13
Mean CC													–0·242	0·058	0·08	0·09	–0·02	0·27*	–0·18
Mean path length														–0·107	–0·05	0·03	0·08	0·05	–0·16
Mean vulnerability															–0·288*	0·10	–0·19	–0·13	0·05

MeDi, Mediterranean diet; DASH, Dietary Approaches to Stop Hypertension; MIND, Mediterranean-DASH Intervention for Neurodegenerative Delay; CC, clustering coefficient; MMSE, Mini-Mental State Examination; RVIP, rapid visual information processing; Acc, accuracy; TR, response time.

Spearman’s correlation coefficient, *P < 0·05, **P < 0·01.

The mu-value demonstrated a negative association with the response time on the RVIP task. The mean clustering coefficient exhibited a positive association with accuracy on the RVIP task. Lastly, mean vulnerability was negatively associated with MMSE.

#### Relationship between age and cognitive outcomes and global metrics

To explore the relationship between age, cognitive outcomes and global metrics, a hierarchical regression analysis was conducted while controlling for years of education and BMI (refer to Tables 11 and 12). The tables present the regression coefficients and standard errors for each analysis. Elevated age demonstrated a significant association with reduced contextual memory (*P* = 0·001), which remained significant even after correcting for multiple comparisons using the Benjamini and Hochberg method^(51)^ (see Table 11). Additionally, increased age exhibited associations with lower mu-value (*P* = 0·002) and modularity (*P* = 0·042) as presented in Table 12. However, after correction for multiple comparisons, only the association with mu-value remained statistically significant.

**Table 11.** Association of age with cognitive scores, standardised coefficients beta and adjusted R-square

	MMSE		Spatial working memory		Contextual memory		RVIP Acc		RVIP TR	
	*β*	R^2^	*β*	R^2^	*β*	R^2^	*β*	R^2^	*β*	R^2^
Age	−0·218	0·055	−0·102	−0·048	−0·441*** #	0·151	−0·022	−0·048	0·1	−0·051

MMSE, Mini-Mental State Examination; RVIP, rapid visual information processing; Acc, accuracy; TR, response time.

Hierarchical regressions controlling for years of education and BMI. # Significant with false discovery rate correction, ***P < 0·001.

**Table 12.** Association of age with graph theory metrics, standardised coefficients beta and adjusted R-square

	Mu value		Modularity		Assortativity		Small worldness		Normalised CC		Normalised path length		Global efficiency		Mean local efficiency		Mean CC		Mean path length		Mean vulnerability	
	*β*	R^2^	*β*	R^2^	*β*	R^2^	*β*	R^2^	*β*	R^2^	*β*	R^2^	*β*	R^2^	*β*	R^2^	*β*	R^2^	*β*	R^2^	*β*	R^2^
Age	−0·414** #	0·163	−0·268*	0·111	0·127	−0·022	0·264^	0·053	0·108	−0·038	−0·176	0·009	−0·059	−0·038	0·102	−0·016	−0·194	−0·015	−0·008	0·075	0·256^	0·078

CC, clustering coefficient.

Hierarchical regressions controlling for years of education and BMI. # Significant with false discovery rate correction, *P < 0·05, **P < 0·01, ^*P* < 0·10, #*P* = 0.002.

#### Relationship between dietary pattern scores and cognitive scores

The association between dietary patterns and cognitive outcomes was examined using hierarchical regressions while accounting for age, years of education and BMI as control variables (refer to Table 13). The analysis did not reveal any significant relationships between dietary patterns and MMSE, spatial working memory, contextual memory or RVIP.

**Table 13.** Association of dietary pattern scores with cognitive scores, standardised coefficients beta and adjusted R-square

	MMSE		Spatial working memory		Contextual memory		RVIP Acc		RVIP TR	
	*β*	R^2^	*β*	R^2^	*β*	R^2^	*β*	R^2^	*β*	R^2^
MeDi	–0·19	0·06	–0·002	–0·039	–0·028	0·111	0·025	–0·077	–0·148	–0·055
DASH	–0·258^	0·087	–0·164	–0·014	0·019	0·111	–0·107	–0·066	0·03	–0·076
MIND	–0·239^	0·082	–0·237	0·018	0·225^	0·162	–0·241	–0·019	–0·081	–0·071

MMSE, Mini-Mental State Examination; RVIP, rapid visual information processing; Acc, accuracy; TR, response time; MeDi, Mediterranean diet; DASH, Dietary Approaches to Stop Hypertension; MIND, Mediterranean-DASH Intervention for Neurodegenerative Delay.

Hierarchical regressions controlling for age, years of education and BMI. ^*P* < 0·10.

#### Relationship between dietary patterns and global brain metrics

The hierarchical regressions, performed to investigate the association between dietary patterns and global brain metrics, revealed that higher adherence to the MeDi was associated with lower assortativity and an increased mean path length (Table 14). Further, higher adherence to the DASH diet showed an association with higher small-worldness. However, after controlling for multiple comparisons, none of these associations remained statistically significant.

**Table 14.** Association of dietary pattern scores with graph theory metrics, standardised coefficients beta and adjusted R-square

	Mu value		Modularity		Assortativity		Small worldness		Normalised CC		Normalised path length		Global efficiency		Mean local efficiency		Mean CC		Mean path length		Mean vulnerability	
	*β*	R^2^	*β*	R^2^	*β*	R^2^	*β*	R^2^	*β*	R^2^	*β*	R^2^	*β*	R^2^	*β*	R^2^	*β*	R^2^	*β*	R^2^	*β*	R^2^
MeDi	0·062	0·095	0·201	0·083	−0·316*	0·02	−0·022	0·059	0·064	−0·043	0·156	−0·014	−0·175	−0·007	−0·212	0·01	0·225	0·027	0·313*	0·17	0·018	0·03
DASH	−0·061	0·094	0·11	0·054	−0·108	−0·071	0·288*	0·138	0·285^	0·031	0·114	−0·026	−0·171	−0·01	−0·278^	0·039	−0·153	−0·002	0·133	0·087	0·15	0·051
MIND	0·052	0·094	0·139	0·062	−0·193	−0·044	0·157	0·083	0·076	−0·041	−0·084	−0·031	−0·139	−0·019	−0·144	−0·014	−0·105	−0·013	0·112	0·083	0·072	0·035

CC, clustering coefficient; MeDi, Mediterranean diet; DASH, Dietary Approaches to Stop Hypertension; MIND, Mediterranean-DASH Intervention for Neurodegenerative Delay.

Hierarchical regressions controlling for age, years of education and BMI. None yielded significant results after false discovery rate correction. *P < 0·05, ^*P* < 0·10.

#### Relationship between dietary pattern scores and network-based statistics/threshold-free network-based statistics outcomes

In the SCD cohort, significantly reduced connectivity emerged between two nodes (right lateral occipital to right precuneus) in the primary NBS analysis. This analysis, which set a threshold of 3·5 and a density mask of 30 %, compared the group with the lowest adherence to the DASH diet against the middle tertile adherence group. No further differences emerged when comparing the MeDi or MIND adherence groups.

**Figure 3.**
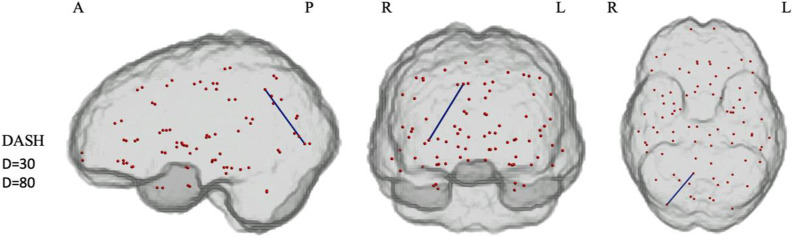
Subnetworks with reduced connectivity in individuals adhering to the lowest tertile of the DASH dietary pattern compared with individuals in the middle tertile group of the DASH dietary pattern in the subjective cognitive decline cohort. Weakened connections are highlighted in blue edges, and nodes are presented in red, which are all equal-sized. This analysis is based on the sensitivity analysis with a 30 and 80 % density mask and presents the results of threshold 3·5 (controlled for covariates age, BMI and year of education). DASH, Dietary Approaches to Stop Hypertension; A, anterior; L, left hemisphere; P, posterior; R, right hemisphere.

Further sensitivity analysis, which involved increasing the density mask to 80 %, confirmed the previously found connectivity difference (Fig. 3 and Table 15). However, when lowering the thresholds to 3 or 2·38 and increasing density to 100 %, no differences were found.

**Table 15.** Connectivity differences between low adherence and middle tertile adherence in the DASH dietary patterns

Dietary pattern	Density mask	Nodes		t	*P*
DASH	30 80	59–73	Right lateral occipital to right precuneus	3·79	0·02

DASH, Dietary Approaches to Stop Hypertension.

This table displays the *t*-value and corresponding *P*-values from the network-based statistics analysis. The analysis applied a statistical significance level of *P* = 0·05 (threshold = 3·5, one-tailed *t* test) to identify edges exceeding the threshold within interconnected components. The statistical significance of each detected component was assessed using an empirical null distribution derived through 5000 permutations.

The TFNBS analysis did not show any significant differences.

## Discussion

The present study aimed to evaluate the association between the three dietary patterns – the MeDi, DASH and MIND – and cognitive outcomes and white matter connectivity in both an HMA and a cohort of older adults with SCD. The study found that age played a significant role, inversely correlated with certain cognitive measures and brain metrics, such as contextual memory and mu-value, across both cohorts. Despite the established benefits of the MeDi, DASH and MIND diets for cardiovascular and neurodegenerative diseases, our study did not reveal an association between these dietary patterns and optimal cognitive outcomes or altered global brain metrics after adjusting for multiple comparisons. This suggests that adherence to these dietary patterns does not account for a large proportion of the age-associated changes to cognition and white matter connectivity observed. In the older SCD population, individuals with lower adherence to the DASH diet exhibited reduced connectivity in the right hemisphere, as demonstrated by the NBS analysis. The HMA cohort, which included a younger, cognitively healthy population, also showed lower white matter connectivity with reduced adherence to the MIND and DASH diet, but these findings did not remain consistent across different density masks or thresholds. Additionally, this study employed TFNBS, an approach less dependent on thresholds, although not entirely independent of setting parameters. While using conservative parameters, the present study observed no differences between the dietary adherence groups, raising questions about the NBS findings’ reliability.

The findings of the present study are in contrast with Rodrigues *et al.* (2020), which reported significant cognitive benefits linked to adherence to the MeDi – specifically within subnetworks governing sensory stimuli and reward integration, including regions such as the olfactory cortex, amygdala, calcarine, lingual and middle occipital gyri – our present study did not observe similar trends. This discrepancy could be indicative of the distinct populations examined and the methodological techniques employed in the respective studies, as well as the varying degrees of MeDi adherence being compared. When assessing the DASH and MIND diet, findings across the two cohorts were not consistent. Specifically, the older SCD cohort showed reduced connectivity from the right lateral occipital cortex to the right precuneus. The right lateral occipital cortex is involved in visual processing, while the right precuneus is crucial for cognitive functions such as memory. Reduced connectivity between these regions, observed in the low DASH adherence group, can indicate cognitive decline, as both areas are integral to higher-order cognitive processes and spatial integration. In contrast, the HMA cohort displayed decreased connectivity across two interhemispheric regions. Similarly, lower adherence to the MIND diet was associated with reduced connectivity between the left supramarginal and left transverse temporal regions. To clarify, the left supramarginal gyrus is involved in processing sensory information and integrating it with motor functions, while the left transverse temporal gyrus plays a role in auditory processing. Reduced connectivity between these regions suggests a potential disruption in integrating sensory and auditory information, potentially affecting cognitive functions. These findings underscore the need for future, large-scale studies to validate the influence of the MIND and DASH diets on neural connectivity as this is the first study assessing these dietary patterns on white matter connectivity. A noteworthy observation from this study is the correlation between the mu-value and age, suggesting that this measure may be a valuable addition to future studies investigating age-related changes in the connectome. Previous research has established a correlation between the MIND diet and various cognitive health outcomes^(9,52)^. Other studies have found an association with subjective cognitive complaints, a decreased likelihood of developing cognitive impairment, mild cognitive impairment and dementia, as well as improved cognitive function in those with high adherence – the associations with the DASH diet have been inconsistent^(9)^. Although most measures changed as anticipated, our investigation did not reveal any significant differences across the dietary pattern groups, cognitive outcomes or white matter connectivity measures, contrary to earlier studies that suggested a clear link between these dietary patterns and cognitive measures. It is plausible that increasing the sample size in future studies could reveal more subtle effect sizes that were not detected in the present study’s cohorts.

The present study’s strength lies in the application of advanced analysis methods, including NBS and TFNBS, to examine structural connectivity. In contrast to previous research by Rodrigues *et al.* (2020), which utilised single-shell data, this study utilised multi-shell methods to construct the connectome, enhancing the accuracy of structural connectivity assessment. Additionally, precise techniques such as distortion correction and anatomically constrained tractography were employed to address concerns about streamlines’ accuracy and frontal cortex mapping distortions. The integration of SIFT2 further improved the accuracy of structural connectivity analysis, reducing biases in probabilistic tractography. This advanced imaging technique enhances the accuracy and precision of structural connectivity assessment. An additional strength of the paper is the inclusion of two study cohorts along the continuum of cognitive ageing, which is a novel approach to study these associations, in seeking out robust associations that replicate across such samples. Another strength of the present study is the inclusion of a study population that used diverse inclusion criteria to ensure a diverse representation of diet quality, with 50 % adhering to an ‘optimal’ diet and the remaining 50 % following a suboptimal diet as assessed by the Diet Screening Tool^(23)^. Furthermore, our adherence to the detailed dietary scoring methodology as recommended by Arnoldy *et al.* (2024) provides a standardised approach to dietary pattern scoring, which lessens subjectivity and enhances reproducibility across research studies. Another strength of the present study is the inclusion of multiple dietary patterns instead of only assessing the MeDi.

This study recognises several limitations that need careful consideration. First, the cross-sectional design restricts our ability to establish causal relationships between dietary patterns and health outcomes. To provide deeper insights into these associations, future research should prioritise prospective longitudinal approaches. Another methodological limitation comes from the varied dietary assessment methods utilised across the two cohorts. Where the HMA cohorts utilised ASA24 the SCD cohort used the CCV FFQ, both with a varied number of included items. Additionally, the unavailability of data on olive oil consumption in the CCV FFQ resulted in a reduction in the maximum achievable scores for the MeDi and MIND diets and their underestimation of adherence scores for these dietary patterns, potentially diminishing the accuracy of the findings of the SCD cohort. Further complicating the dietary assessment was the stringent scoring protocol for the MIND diet, which assigned a score of 1 to individuals consuming a precise serving of alcohol equivalent to 5 ounces. This inflexibility may fail to accurately capture variations in alcohol intake, potentially leading to misrepresentation of adherence levels. Additionally, while we employed continuous scores for the regression, the use of data-driven adherence levels was used in the NBS and TFNBS analysis, which presents limitations. While literature-based adherence levels might enhance cross-sectional comparability, applying literature-based adherence levels was not feasible in our datasets. The MeDi adherence levels were in both cohorts lower than the definition of high adherence described in the original method paper by Martinez-Gonzalez *et al.* (2012) – where individuals in the highest tertile had scores of at least 10 – the individuals in our study did not surpass the first two tertiles with a maximum score of 6 in the SCD cohort and 9 in the HMA cohort. This may be influenced by geographical variations and documented low adherence to vegetables in Australian populations^(53)^. This discrepancy could also explain the lack of findings found in the present paper compared with the paper by Rodrigues *et al.* (2020), who compared individuals with a MeDi score of 10 to individuals with a MeDi score below 10. The data-driven adherence levels for the DASH and MIND diets in the present study were similar to those reported in the original methodological papers, which may account for the observed decreased connectivity in these diets but not for the MeDi, given the insufficient adherence levels. Moreover, the spread of dietary patterns adherence scores in the SCD dataset was limited, contrasting with the intentional recruitment strategy in the HMA cohort, which might be a reason why no differences were found in the SCD cohort. Additionally, while our sample size was adequate for the primary analyses, research has shown that case–control studies examining network metrics typically require at least sixty-five participants per group to achieve 80 % power when examining single graph metrics^(47)^. Given that standard power analyses are not readily applicable to complex network analyses, this work should be considered exploratory in nature, providing foundations for future confirmatory studies. Future investigations should leverage large-scale databases such as the UK Biobank to validate these findings and identify the most relevant metrics for aging populations. The integration of graph theory metrics (GTM) with network-based statistical approaches (NBS/TFNBS) could provide complementary insights into how lifestyle factors influence brain structure at varying levels of detail. Furthermore, assessing self-reported dietary recall in participants with memory issues introduces potential recall bias and inaccuracies in reported dietary information, further complicating the interpretation of results. Collectively, these limitations necessitate a cautious interpretation of our findings. They serve to underscore the inherent complexities of dietary research and highlight the need for refined assessment tools, more representative sampling methods and longitudinal designs in future studies to bolster the evidence base concerning the impact of diet on cognitive health and brain connectivity.

In conclusion, while our study observed age-associated declines in cognitive functions, the anticipated links between dietary patterns and cognitive outcomes or global brain metrics were not consistently substantiated. Future investigations should prioritise longitudinal designs, improved dietary assessment tools and consideration of geographical and cultural influences on diet to build upon the findings presented here.

## Supporting information

Arnoldy et al. supplementary material Arnoldy et al. supplementary material

## Supplementary material

For supplementary material/s referred to in this article, please visit https://doi.org/10.1017/S0007114525000406.
