# Supplementary material for: Assessing the association between the Mediterranean, Dietary Approaches to Stop Hypertension and Mediterranean-DASH Intervention for Neurodegenerative Delay dietary patterns, structural connectivity and cognitive function
Source: Br J Nutr. 2025 Feb 28;133(7):901–17. doi: 10.1017/S0007114525000406 (PMC12198342; doi:10.1017/S0007114525000406)
Supplement: Arnoldy et al. supplementary material [file S0007114525000406sup001.docx]

#### Supplementary material Section A

| Table 1: Includes the food items extracted from the ASA24 (which also includes the AUSNUT codes), and CCV FFQ for the MeDi dietary pattern. | | | | | |  |  |
| --- | --- | --- | --- | --- | --- | --- | --- |
|  |  | **ASA24** | |  | **CCV FFQ** | |  |
| **MeDi Questions** |  | **Included items (serving size in gram)** | **AUSNUT codes** |  | **Included items (serving size in grams)** | |  |
| **1. Do you use olive oil as main culinary fat?** |  | Olive oil, Plant oils (%) | 14402007, 14 |  | NA | |  |
| **2. How much olive oil do you consume in a given day (including oil used for frying, salads, out of house meals etc?)** |  | Olive oil (13.5) | 14402007 |  | NA | | |
| **3. How many vegetable servings do you consume per day?** |  | Bok choy, Brussels sprouts, Cabbage, Kale, Kohlrabi, Broccoli, Cauliflower, Carrot, Artichoke, Beetroot, Cassava, Celeriac, Chicory, Ginger, Parsnip, Radish, Swede, Taro, Turnip, Wasabi, Endive, Lettice, mixed leafy greens, Rocket, Silver beet, Spinach, Vine leaf, Watercress, Asparagus, bamboo, Celery,  Basil, chives, Coriander, Dill, Flower, Herbs, Mint, Parsley, Rosemary, Tomato, Pumpkin, Squash, Zucchini, Mushroom, Sweetcorn, Avocado, Capsicum, Chilli, Choko, Cucumber, Eggplant, Melon, Okra, Fennel, Garlic, Leek, Onion, Shallot, Seaweeds,  Potatoes, Wild harvested vegetables, Vegetable juice, Fruit/ vegetable juice blends, vegetable- based pickles (Gherkin, ginger, olive, pickles, relish), Vegetable dips (200) | 11305, 11306, 23202, 23502, 24001, 24101, 242-244, 246-248 |  | Avocado, Potatoes (not fat), Tomato sauce, Tomatoes, Capsicum, Lettuce, Cucumber, Celery, Beetroot, Carrots, Cabbage, Cauliflower, Broccoli, Spinach, Peas, Bean sprouts, Pumpkin, Onion, Garlic, Mushrooms, Zucchini (200) | |  |
| **4 How many fruit units (including natural fruit juices) do you consume per day?** |  | Wild harvested fruits, Apple, Pear, Loquat, Quince, Blackberry, Cranberry, Mulberry, Raspberry, Strawberry, Orange, Lemon, Lime, Cumquat, Grapefruit, Mandarin, Tangelo, Tangerine, Mandarin, Nectarine, Peach, Apricot, Cherry, Plum, Banana, Pineapple, Babaco, Cheese fruit, Fig, Persimmon, Tamarillo, Wax jambul, Feijoa, Guava, Jackfruit, Lychee, Mango, Passionfruit, Pawpaw, Pomegranate, Prickly pear, Rambutan, Grape, Kiwifruit, Melon, Pepino, Rhubarb, Quandong, Currant, Raisin, Sultana, Fruit juices (freshly-squeezed) (150) | 11302, 16 |  | Tinned fruit, Oranges, Apples, Pears, Bananas, Melon, Pineapple, Strawberries, Apricots, Peaches, Mango (150) | |  |
| **5 How many servings of red meat, hamburger, or meat products (ham, sausage, etc.) do you consume per day?** |  | Unprocessed Beef, lamb, mutton, pork, veal, kangaroo, buffalo, camel, goat, rabbit, venison, Sausages, Frankfurt’s and saveloys, bacon, ham, prosciutto, kabana, salami, chorizo, mortadella, jerky, Berliner, Devon, Wild harvested mammalian meat (100) | 18011, 181, 182, 185, 186 |  | Beef, Veal, Lamb, Pork, Bacon, Ham, Salami, Sausages, Hamburger (100) | |  |
| **6 How many servings of butter, margarine, or cream do you consume per day?** |  | Butters, Margarine and table spreads, Dairy blends, Unspecified dairy-based fat or margarine used as a spread, Ghee, Cream, Dairy based savoury sauces (12) | 141-143, 14601, 193, 23108 |  | Full cream milk, Margarine, Polyunsaturated margarine, Monounsaturated margarine, Butter and margarine blends, Butter (12) | |  |
| **7 How many sweet/carbonated beverages do you drink per day?** |  | Fruit juice/ drink, Cordial, soft drink, sweetened caffeinated colas, sugar-sweetened caffeine-free colas, other sugar-sweetened carbonated drinks, lemonade or other noncarbonated fruit drink, electrolyte drinks, energy drinks (Times/day) | 11302, 11307, 11309, 114-116, 118 |  | Fruit juice (Times/day) | |  |
| **8 How much wine do you drink per week?** |  | Red and white wine (including sparkling varieties and rose styles), Fortified wines, Reduced alcohol wines (150ml) | 29201-29204 |  | Red wine, white wine, fortifies wine (150ml) | |  |
| **9 How many servings of legumes do you consume per week?** |  | Broad bean, Butter bean, green bean, red bean, black bean, haricot bean, lima bean, lupin bean, red kidney bean, soybean, cannellini bean, baked beans, sprouts, chickpea, lentil, pea, snow pea, tempeh, tofu, Legume and pulse products, Legume based dips (hummus), lentil or other legumes soup (150) | 20601006, 23503, 245, 251, 25201 |  | Green beans, Baked beans, Tofu, Other beans (150) | |  |
| **10 How many servings of fish or shellfish do you consume per week?** |  | Fin fish (Barramundi, Bassa, blue grenadier, blue-eye trevalla, bream, cod, flathead, flounder, gemfish, grouper, john dory, ling, mackerel, milkfish, moronga, mullet, mulloway, Nile perch, Orange, Salmon, Sardine, Shark, Silver perch, Snapper, Swordfish, Tilapia, kingfish, Trout, Tuna, Whitebait, Whiting, caviar, eel, Anchovy, Herring) (125), Wild caught fish and seafood (125), Crab (200), Lobster (200), Moreton bay bug (200), Prawn (200), Mussel (200), Octopus, Oyster (200), Scallop (200), Squid or calamari (200) | 151-154, 15501001, 15501003, 15501004, 15501008, 15501011, 15501014-15501016, 15501019, 15501020, 15501022-15501024, 15501027, 15501028, 15501030-15501033, 15501035, 15501036, 15501038, 15502001-15502003, 157 |  | Fish (125), Tinned fish (125), Fried fish (125) | |  |
| **11 How many times per week do you consume commercial sweets or pastries (not homemade), such as cakes, cookies, biscuits, or custard?** |  | Sweet bread, buns and scrolls, sweet biscuits, cakes, muffins, scones, Waffles, Doughnut, Crumpets, cake-type desserts, plain and sweet Pastry, pancakes, crepes, ice cream, Gelato, Custards, dairy desserts, rice pudding, cheesecake, trifle, tiramisu, Jelly, pavlova, meringue, Chocolate, confectionery (Times/week) | 12305, 12306, 131, 133, 13401-13403, 136, 196, 273, 281, 28202, 284 |  | Jam, Honey, Ice-cream, Chocolate, Flavoured Milk, Sweet Biscuits, Cakes (Times/week) | |  |
| **12 How many servings of nuts (including peanuts) do you consume per week?** |  | Nuts and seeds (30) | 22, 16803 |  | Nuts (30) | |  |
| **13 Do you preferentially consume chicken, turkey, or rabbit meat instead of veal, pork, hamburger or sausage?** |  | Chicken, Duck, turkey, emu, Mutton-bird, Ostrich, Pigeon, Quail (%) | 183 |  | Chicken, Beef, Veal, Chicken, Lamb, Pork, Bacon, Ham, Salami, Sausages | |  |
| **14 How many times per week do you consume vegetables, pasta, rice, or other dishes seasoned with sofrito?** |  | Tomato based products and dishes and tomato-based sauces (not tomato sauce aka ketchup) (Times/week) | 23104, 23106 |  | Tomato sauce (Times/week) | |  |
| **Number of components included in score** |  |  | **14** |  | **12** | |  |
| *Note.* This table presents the included items from the ASA24 and CCV FFQ in the MeDi and presents the grams equivalent to a serving size of each item utilised for scoring the MeDi, indicated in brackets. Abbreviations: ASA24= Automated Self-Administered 24-Hour Dietary Assessment Tool, CCV=Cancer Council, FFQ=Food Frequency Questionnaire, Medi= Mediterranean diet. * Only whole grains: wholemeal, mixed grains, rye, cereal flours, spelt, cornflour | | | | | | |  |

| *Table 2: Includes the food items extracted from the ASA24 (which also includes the AUSNUT codes), and CCV FFQ FFQ for the DASH dietary pattern.* | | | | | |  |
| --- | --- | --- | --- | --- | --- | --- |
|  |  | **ASA24** | |  | **CCV FFQ** |  |
| **DASH Components** |  | **Included items (serving size in grams)** | **AUSNUT codes** |  | **Included items (serving size in grams)** |  |
| **Total Grain intake** |  | Grains for bread (43), cooked Barley (100), Buckwheat groats (100), Bulgur (100), Cornmeal cooked (100) uncooked (57), Oats (uncooked) (57), Quinoa (100), rice (100), Rye cooked (100) uncooked (57), Spelt uncooked (57), Couscous cooked (100) uncooked (57), flour (Semolina, cornflour, rye ,spelt, wholemeal) (57), Tapioca, flatbread (43), muffins (43), Noodles cooked (100), Pasta (100), Breakfast cereal (26), Muesli (57), Porridge (110), Cakes (57), scones (57), cake-type desserts (57), Savoury biscuits (57), Pancakes (57), crepes (57), Waffles (57), popcorn (57), muesli or cereal bars (57), crumpets (57) | 12, 12514, 12515, 132, 133, 13601-13603, 13606, 26202, 283 |  | High fibre white bread (43), White bread (43), Wholemeal bread (43), Rye bread (43), Multi grain bread (43), All bran (26), Brand flakes (26), Weet Bix (26), Cornflakes (26), Porridge (110), Muesli (57), Rice (100), Pasta (100), Crackers (57), Sweet Biscuits (57), Cakes (57) |  |
| **Whole grain intake** |  | Whole grain bread (43), muffin (57), Noodles cooked (100), pasta (100), brown/red Rice (100), Whole grain cold breakfast cereal (26), hot porridge (110), Savoury biscuits (57), Muesli and cereal style bars (57), cooked Barley (100), Bulgur (100), Cornmeal cooked (100), Millet, Oats (uncooked) (57), Quinoa cooked (100), Couscous cooked (100) uncooked (57), Tapioca, whole grain flour (57), popcorn (57) | 12101001-12101003, 12101006-12101011, 12101014-12101022, 12101025, 12101026, 12101030, 12102007-12102009, 12102014, 12103001-12103003, 12103006, 12103007, 12103010, 12103011, 12103014, 12103015, 12103021, 12103022, 12201009, 12201010, 12203013, 12203014, 12204-12212, 12214001, 12301004, 12301005, 12302004, 12302005, 12302008-12302011,12303, 12401018-12401020, 12402, 12403002, 12403007, 12502, 12505, 12506, 12511, 12512, 12514, 12515, 12516001, 13201001, 13201002, 13201008-13201012, 13203, 13204001-13204003, 13205001, 13205002, 12403006, 12501, 12507, 12513,  126, 26202, 28301 |  | Wholemeal bread (43), Rye bread (43), Multi grain bread (43), Brand flakes (26), Weet Bix (26), Porridge (100), Muesli (57) |  |
| **Vegetables** |  | Endivev (35), lettuce (35), mixed leafy greens (35), rocket (35), silver beet (35), spinach (35), nine leaf (35), watercress (35), Bok choy (35), brussels sprout (35), cabbage (35), kale (35), kohlrabi (35), Potatoes (70), Tomato and tomato products (70), pumpkin (70), Squash and zucchini (70), Mushrooms (70), Sweetcorn (70), avocado (70), capsicum (70), chilli (70), choke (70), cucumber (70), eggplant (70), melon (70), okra (70), fennel (70), water chestnut (70), garlic (70), leek (70), onion (70), Shallot (70), broccoli (70), Broccolini (70), Cauliflower (70), Wild harvested vegetables (70), peas (70), sprouts (70), carrot (70), beetroot (70), cassava (70), celeriac (70), ginger (70), radish (70), chicory (70), Swede (70), Taro (70), Turnip (70), Wasabi (70), artichoke (70), asparagus (70), bamboo shoot (70), celery (70), Wild harvested (70), green/ snow peas (70), alfalfa sprouts (70), bean sprouts (70), freshly squeezed vegetable juices (120), Fruit and vegetable juice blends (120), Vegetable-based pickles (70), chutneys (70), relishes and dips (70) | 11305, 11306, 23104, 23106, 23202, 23502, 24001, 24101, 242-244, 246-248, 24501, 24503, |  | Avocado (70), Potatoes (70), Tomato sauce (70), Tomatoes (70), Capsicum (70), Lettuce (35), Cucumber (70), Celery (70), Beetroot (70), Carrots (70), Cabbage (35), Cauliflower (70), Broccoli (70), Spinach (35), Peas (70), Bean sprouts (70), Pumpkin (70), Onion (70), Garlic (70), Mushrooms (70), Zucchini (70) |  |
| **Fruits** |  | Fruits (Wild harvested fruits, Apple, Pear, Loquat, Quince, Berries, Orange, Lemon, Lime, Cumquat, Grapefruit, Mandarin, Tangelo, Tangerine, Mandarin, Nectarine, Peach, Apricot, Cherry, Plum, Banana, Pineapple, Babaco, Cheese fruit, Fig, Persimmon, Tamarillo, Wax jambul, Feijoa, Guava, Jackfruit, Lychee, Mango, Passionfruit, Pawpaw, Pomegranate, Prickly pear, Rambutan, Grape, Kiwifruit, Melon, Pepino, Rhubarb, Plum, Quandong, Currant, Raisin, Sultana) (150*), Fruit juices (freshly-squeezed) (124) | 16, 11302 |  | Tinned fruit (113), Fruit juice (124), Oranges (150), Apples (182), Pears (150), Bananas (118), Melon (150), Pineapple (150), Strawberries (67), Apricots (150), Peaches (150), Mango (150) |  |
| **Dairy** |  | Cow and goat milk (246), yoghurt (246), cream (214*), blue vein cheese (42.5), cheddar (42.5), Cheshire (42.5), Colby style (42.5), edam (42.5), fetta (42.5), goat cheese (42.5), Gloucester style (42.5), gouda, (42.5) haloumi (42.5), Havarti style (42.5), Jarlsberg (42.5), mozzarella (42.5), parmesan (42.5), pecorino (42.5), provolone (42.5), Romano (42.5), Swiss (42.5), bocconcini (42.5), cottage (42.5), cream cheese (42.5), Neufchatel (42.5), ricotta (42.5), brie (42.5), camembert (42.5), ice cream (246), Frozen Yoghurt (246), Sundae, Custard (246), Dairy dessert (246), Formia’s frais (246), Pudding (246), cheesecake (246), Trifle (246), Tiramisu (246), Iced coffee (246), Milkshake (246), Thick shake (246), Milk-based fruit drinks (246), dairy based savoury sauces (214*), Dairy based dips (214*) | 181, 182, 185, 186, 18011 |  | Full cream milk (246), Reduced fat milk (246), Skim milk (246), Hard cheese (42.5), Firm cheese (42.5), soft cheese (42.5), Ricotta or cottage cheese (42.5), Cream cheese (42.5), Low fat cheese (42.5), Flavoured Milk Drinks (246), Ice cream (148), Yoghurt (246) |  |
| **Meats, poultry, and fish** |  | Unprocessed Beef (85), lamb (85), mutton (85), pork (85), veal (85), kangaroo(85) , buffalo (85), camel (85), goat (85), rabbit (85), venison (85), Sausages (85), Frankfurt’s and saveloys (85), bacon (85), ham (85), prosciutto (85), kabana (85), salami (85), chorizo (85), mortadella (85), jerky (85), Wild harvested mammalian meat (85), Chicken (85), Duck (85), turkey (85), emu (85), Mutton-bird (85), Ostrich (85), Pigeon (85), Quail (85), Barramundi (85), bass (85), blue grenadier (85), blue-eye trevalla (85), bream (85), cod (85), flathead (85), flounder (85), gemfish (85), grouper (85), john dory (85), ling (85), mackerel (85), milkfish (85), moronga (85), mullet (85), mulloway (85), Nile perch (85), Orange (85), Salmon (85), Sardine (85), Shark (85), Silver perch (85), Snapper (85), Swordfish (85), Tilapia (85), kingfish (85), Trout (85), Tuna (85), Whitebait (85), Whiting (85), caviar (85), eel (85), Anchovy (85), Herring (85), Wild caught fish and seafood (85), Crab (85), Lobster (85), Moreton bay bug (85), Prawn (85), Mussel (85), Octopus (85), Oyster (85), Scallop (85), Squid or calamari (85), Egg (50), Wild harvested eggs (50), savoury egg dishes (50) | 151, 153, 154, 157 |  | Beef (85), Veal (85), Chicken (85), Lamb (85), Pork (85), Bacon (85), Ham (85), Salami (85), Sausages (85), Fish (85), Fried fish (85), Tinned fish (85), Eggs (50) |  |
| **Nuts, seeds, and dry beans** |  | Dried fruit and nut mixes (42.5), Tempeh (98), Tofu (98), Lentil/ legumes soup (98), seeds (chi, linseed, flaxseed, poopy, pumpkin, sesame, sunflower, acacia, psyllium, tahini) (42.5), Nuts (Peanuts and products, Coconut, almond, cashew chestnut hazelnut, macadamia, pecan, pine, pistachio, walnut, pandanus, Brazil) (42.5), wild harvested seeds and nuts (42.5), Hummus (98), Beans (black, haricot, lima, lupin, red kidney, soya, cannellini, lupin) (98), chickpea (98), lentil (98), pea (98), Legume and pulse products (miso, chick pea/ soy flour, baked beans) (98) | 183, 18903001, 18903002, 18903005-18903011, 18903014-18903023, 18903048-18903054 |  | Nuts, Peanut butter (42.5) |  |
| **% kcal from fat** |  | Total energy, fat | 20601006-20601012, 21602001, 23503, 24502, 251, 25201 |  | Total energy, fat |  |
| **% kcal from saturated fatty acids** |  | Total energy, saturated fat | 22, 16803 |  | Total energy, saturated fat |  |
| **Sweets** |  | sweetened caffeinated colas (372), sugar-sweetened caffeine-free colas (372), other sugar-sweetened carbonated drinks (372), lemonade or other noncarbonated fruit drink (372), electrolyte drinks (372), energy drinks (372), sweet bread (100), buns and scrolls (100), sweet biscuits (100), cakes (100), muffins (100), scones (100), cake-type desserts (100), plain and sweet Pastry (100), pancakes (100), crepes (100), Waffles (100), Doughnut (100), Crumpets (100), ice cream, Gelato (119), Custards (100), dairy desserts (100), rice pudding (100), cheesecake (100), trifle (100), tiramisu (100), milkshake (100), thick shake, iced coffee (119), Iced chocolate, sugar (18), honey and sugar syrups (18), toppings (18), icing (18), sweet spreads (18), Jelly (18), pavlova (100), meringue (100), Chocolate (100), | 12307, 15502004, 24102, 15501002, 15501006, 15501007, 15501010, 15501012, 15501013, 15501017, 15501018, 15501021, 15501026, 15501029, 15501034, 15501037, 15501039, 15501040, 18903025-18903047, 18903012, 18903013, 13501, 13502, 13503037-13503052, 13505-13508, 136, 26 |  | Sugars (18), Jam (18), Ice cream (100), Chocolate (100), Flavoured Milk Drinks (100), Sweet Biscuits (100), Cakes (100) |  |
| **Sodium** |  | Sodium (mg/day) | 14402007 |  | Sodium (mg/day) |  |
| **Number of components included in score** |  |  | **11** |  | **11** |  |
| *Note.* This table presents the included items from the ASA24 and CCV FFQ in the DASH diet and presents the grams equivalent to a serving size of each item utilised for scoring the DASH, indicated in brackets. Abbreviations: ASA24= Automated Self-Administered 24-Hour Dietary Assessment Tool, CCV=Cancer Council, DASH= Dietary Approaches to Stop Hypertension, FFQ=Food Frequency Questionnaire. *= average | | | | | | |

| Table 3: Includes the food items extracted from the ASA24 (which also includes the AUSNUT codes) and CCV FFQ for the MIND. | | | | | | |
| --- | --- | --- | --- | --- | --- | --- |
|  |  | **ASA24** | |  | **CCV FFQ** |  |
| **MIND Components** |  | **Included items (serving size in grams)** | **AUSNUT codes** |  | **Included items (serving size in grams)** |  |
| **Whole grains*** |  | Whole grain bread (43), Muesli (38), Noodles cooked (133) uncooked (38), pasta cooked (133) uncooked (38), brown/red Rice cooked (133) uncooked (38), Whole grain cold breakfast cereal (38), hot porridge (180), Savoury biscuits (57), Muesli and cereal style bars (57), Barley cooked (133) uncooked (38), Bulgur cooked (133) uncooked (38), Cornmeal cooked (133), Millet cooked (133), Oats uncooked (57), Quinoa cooked (133) uncooked (38), Couscous cooked (133) uncooked (38), Tapioca (133), whole grain flour uncooked (57), popcorn (57) | 12101001-12101003, 12101006-12101011, 12101014-12101022, 12101025, 12101026, 12101030, 12102007-12102009, 12102014, 12103001-12103003, 12103006, 12103007, 12103010, 12103011, 12103014, 12103015, 12103021, 12103022, 12201009, 12201010,  12203013, 12203014, 12204 - 12212, 12214001, 12301004, 12301005, 12302004, 12302005, 12302008 - 12302010, 12302011, 12303, 12401018-12401020, 12402, 12403002, 12403007, 12516001, 12502, 12505, 12506, 12511, 12512, 12514, 12515, 126, 13201001, 13201002, 13201008-13201012, 13203, 13204001, 13204002, 13204003, 13205001, 13205002, 12403006, 12501, 12507, 12513, 26202, 28301 |  | Wholemeal bread (43), Rye bread (43), Multi grain bread (43), Brand flakes (38), Weet Bix (38), Porridge cooked (180), Muesli (38) |  |
| **Green leafy vegetables** |  | Endive, lettuce, mixed leafy greens, rocket, silver beet, spinach, nine leaf, watercress, bock choy, brussels sprout, cabbage, kale, kohlrabi (35) | 24401, 24201 |  | Lettuce, Cabbage, Spinach (35) |  |
| **Other vegetables** |  | Vegetables (Potatoes, Tomato and tomato products, pumpkin, Squash and zucchini, Mushrooms, Sweetcorn, avocado, capsicum, chilli, choke, cucumber, eggplant, melon, okra, fennel, water chestnut, garlic, leek, onion, broccoli, Broccolini, Cauliflower, Wild harvested vegetables, peas, sprouts, carrot, beetroot, cassava, celeriac, ginger, radish, chicory, artichoke, asparagus, bamboo shoot, celery) (70), seaweeds (70), Vegetable-based (pickles, chutneys, relishes and dips) (70), freshly squeezed vegetable juices (120) | 11305, 23104, 23106, 23202, 23502, 24001, 24101, 24202, 243, 24402, 24404, 24501, 24503, 246, 247, 248 |  | Avocado, Potatoes, Tomato sauce, Tomatoes, Capsicum, Cucumber, Celery, Beetroot, Carrots, Cauliflower, Broccoli, Peas, Bean sprouts, Pumpkin, Onion, Garlic, Mushrooms, Zucchini (70) |  |
| **Berries** |  | Blackberry, cranberry, mulberry, raspberry, strawberry, goji berry (67) | 162, 16802005, 16802008, 16802009 |  | Strawberries (67) |  |
| **Red meat + products** |  | Unprocessed Beef, lamb, mutton, pork, veal, kangaroo, buffalo, camel, goat, rabbit, venison, Sausages, Frankfurt’s and saveloys, bacon, ham, prosciutto, kabana, salami, chorizo, mortadella, jerky, Wild harvested mammalian meat (85) | 181, 182, 185, 186, 18011 |  | Beef, Veal, Lamb, Pork, Bacon, Ham, Salami, Sausages (85) |  |
| **Fish** |  | Fin fish (Barramundi, Bassa, blue grenadier, blue-eye trevalla, bream, cod, flathead, flounder, gemfish, grouper, john dory, ling, mackerel, milkfish, moronga, mullet, mulloway, Nile perch, Orange, Salmon, Sardine, Shark, Silver perch, Snapper, Swordfish, Tilapia, kingfish, Trout, Tuna, Whitebait, Whiting, caviar, eel, Anchovy, Herring, Wild caught fish and seafood  (85) | 151, 153, 154, 157 |  | Fish, Tinned fish (85) |  |
| **Poultry** |  | Chicken, Duck, turkey, emu, Mutton-bird, Ostrich, Pigeon, Quail (85) | 183, 18903001, 18903002, 18903005-18903011, 18903014-18903023, 18903048-18903054 |  | Chicken (85) |  |
| **Beans** |  | Bean, black bean, haricot bean, lima bean, lupin bean, red kidney bean, soybean, cannellini bean, baked beans, chickpea, lentil, pea, tempeh, tofu, Legume based dips, lentil or other legumes soup (98) | 20601006-20601012, 21602001, 23503, 24502, 251, 25201 |  | Green beans (98), Baked beans (98), Tofu (98), Other beans (98) |  |
| **Nuts** |  | Nuts and seeds (42.5) | 22, 16803 |  | Nuts (42.5) |  |
| **Fast/fried foods** |  | How often do you eat fried food away from home (like French fries, chicken nuggets (Times/day) | 12307, 15502004, 24102, 15501002, 15501006, 15501007, 15501010, 15501012, 15501013, 15501017, 15501018, 15501021, 15501026, 15501029, 15501034, 15501037, 15501039, 15501040, 18903025-18903047, 18903012, 18903013, 13501, 13502, 13503037-13503052, 13505-13508, 136, 26 |  | Fried fish, Meat pies, Pizza, Hamburger (Times/day) |  |
| **Olive oil** |  | Olive oil consumption was scored 1 if identiﬁed by the participant as the primary oil usually used at home and 0 otherwise (>=50% of average intake: (gr olive oil/gr total fat *100)) | 14402007 |  | NA |  |
| **Butter, margarine** |  | Butters, Margarine and table spreads, Dairy blends, Unspecified dairy-based fat or margarine used as a spread (14) | 141-142, 14601 |  | Margarine, Polyunsaturated margarine, Monounsaturated margarine, Butter and margarine blends, Butter (14) |  |
| **Cheese** |  | Blue vein cheese, cheddar, Cheshire, Colby style, edam, fetta, goat, Gloucester style, gouda, haloumi, Havarti style, Jarlsberg, mozzarella, parmesan, pecorino, provolone, Romano, Swiss, bocconcini, cottage, cream cheese, ricotta, brie, camembert (42.5) | 194 |  | Hard cheese, Firm cheese, soft cheese, Ricotta or cottage cheese, Cream cheese, Low fat cheese (42.5) |  |
| **Pastries, sweets** |  | Sugar (18), Honey and sugar syrups (18), Toppings (18), Jam (18), marmalade (18), Sweet spreads or sauces (18), Jelly (18), Meringue (18), Pavlova (100), sorbet (119), gelato (119), Icing (18), Chocolate/ Chocolate bar (100), Sweet biscuits (100), Cakes (100), muffins (100), scones(100) , cake-type desserts (100), brownie (100), Crepe (100), Pancake (100), Pikelet (100), Waffle (100), Fritter (100), Doughnuts (100), Crumpets (100), Ice cream (199), Frozen Yoghurt (199), Dairy desserts (100), Flavoured milks/milkshakes (152), Caramels (18), Fudge (18), liquorice (18), Lolly (18), Lollipop (18), Marshmallow (18), Sherbet (18), Turkish delight (18), Chewing gum (18), Sweet breads (100), Sweet biscuits (100), Pastry (100), Nut and seed based confectionery (18) | 12305, 12306, 131, 133, 13401-13403, 136, 195-198, 281, 284, 27, 28202 |  | Sugars (18), Jam (18), Ice cream (119), Chocolate (100), Flavoured Milk Drinks (152), Sweet Biscuits (100), Cakes (100) |  |
| **Wine** |  | Red and white wine (including sparkling varieties and rose styles), Fortified wines, Reduced alcohol wines (141.7) | 29201-29204 |  | Red wine, white wine, fortifies wine (141.7) |  |
| **Number of components included in score** |  |  | **15** |  | **14** |  |
| *Note.* This table presents the included items from the ASA24 and CCV FFQ in the MIND and presents the grams equivalent to a serving size of each item utilised for scoring the MIND, indicated in brackets. Abbreviations: ASA24= Automated Self-Administered 24-Hour Dietary Assessment Tool, CCV=Cancer Council, FFQ=Food Frequency Questionnaire, MIND=Mediterranean-DASH Intervention for Neurodegenerative Delay, NA = Not Applicable. | | | | | | |

#### Supplementary material Section B

#### Biochemical assessment

For the biochemical evaluation aspect of the research, the MAST and PLICAR study's blood samples were obtained by a nurse or trained phlebotomist affiliated with Swinburne University. In each study, fasting blood glucose levels were determined through HbA1c measurements. Additionally, blood was tested for certain biomarkers, including homocysteine and high-sensitivity C-reactive protein (HsCRP).

#### Cardiovascular measures

For the PLICAR and MAST study, blood pressure readings were taken after a five-minute rest, with participants lying down. Utilizing the SphygmoCor XCEL, a blood pressure cuff was automatically inflated around the arm to measure brachial blood pressure, and this process was repeated three times. The average of these readings was calculated for the statistical analysis.

#### Supplementary material Section C

#### MRI Data Pre-processing and Connectome Construction

MRI data was pre-processed using QSIPrep (Cieslak et al., 2021) which adheres to the best practice in the field. After initial processing the connectomes for individuals in the MAST and PLICAT dataset were created in an identical fashion utilising QSIPrep (Cieslak et al., 2021) and MRtrix3 (v3.0.3_2021/09/17) (J.-D. Tournier et al., 2019) using Neurodesk (Renton et al., 2024).

##### Anatomical data pre-processing

The T1-weighted (T1w) data was corrected for intensity non-uniformity using N4BiasFieldCorrection (Tustison et al., 2010), and was used as T1w-reference throughout the workflow. The T1w-reference was then skull-stripped using antsBrainExtraction.sh (ANTs 2.4.0), using OASIS as a target template. Spatial normalisation to the ICBM 152 Nonlinear Asymmetrical template version 2009c (Fonov, Evans, McKinstry, Almli, & Collins, 2009) (RRID: SCR_008796) was performed through nonlinear registration with antsRegistration (ANTs 2.4.0, RRID: SCR_004757) (Avants, Epstein, Grossman, & Gee, 2008), using brain-extracted versions of both T1w volume and template. Brain tissue segmentation of cerebrospinal fluid (CSF), white matter (WM) and grey matter (GM) was performed on the brain-extracted T1w using FAST (FSL 6.0.5.1:57b01774, RRID: SCR_002823) (Zhang, Brady, & Smith, 2001). Brain surfaces were reconstructed using recon-all (FreeSurfer 7.2.0, RRID:SCR_001847) (Dale, Fischl, & Sereno, 1999), and the brain mask estimated previously was refined with a custom variation of the method to reconcile ANTs-derived and FreeSurfer-derived segmentations of the cortical gray-matter of Mindboggle (RRID:SCR_002438) (Klein et al., 2017).

##### Diffusion data pre-processing

Any images with a b-value less than 100 s/mm^2 were treated as a b=0 image. MP-PCA denoising as implemented in Mrtrix3’s dwidenoise (Veraart et al. 2016) was applied with a 5-voxel window. After MP-PCA, Gibbs unringing was performed using Mrtrix3’s mrdegibbs (Kellner, Dhital, Kiselev, & Reisert, 2016). Following unringing, B1 field inhomogeneity was corrected using dwibiascorrect from Mrtrix3 with the N4 algorithm (Tustison et al., 2010). After B1 bias correction, the mean intensity of the DWI series was adjusted so all the mean intensity of the b=0 images matched across each separate DWI scanning sequence.

FSL’s (version 6.0.5.1:57b01774) eddy was used for head motion correction and Eddy current correction (Andersson & Sotiropoulos, 2016). Eddy was configured with a q-space smoothing factor of 10, a total of 5 iterations, and 1000 voxels used to estimate hyperparameters. A linear first-level model and a linear second-level model were used to characterize Eddy current-related spatial distortion. Q-space coordinates were forcefully assigned to shells. Field offset was attempted to be separated from subject movement. Shells were aligned post-eddy. Eddy’s outlier replacement was run (Andersson, Graham, Zsoldos, & Sotiropoulos, 2016). Data were grouped by slice, only including values from slices determined to contain at least 250 intracerebral voxels. Groups deviating by more than 4 standard deviations from the prediction had their data replaced with imputed values. Data was collected with reversed phase-encode blips, resulting in pairs of images with distortions going in opposite directions. Here, b=0 reference images with reversed phase encoding directions were used along with an equal number of b=0 images extracted from the DWI scans. In the PLICAR dataset, three reversed phase images were used while in the MAST dataset, only one was used. From these pairs, the susceptibility-induced off-resonance field was estimated using a method similar to that described in (Andersson, Skare, & Ashburner, 2003). The fieldmaps were ultimately incorporated into the Eddy current and head motion correction interpolation. Final interpolation was performed using the jac method.

Several confounding time series were calculated based on the pre-processed DWI: framewise displacement (FD) using the implementation in Nipype (following the definitions by Power et al. 2014 (Power et al., 2014)). The head-motion estimates calculated in the correction step were also placed within the corresponding confounds file. Slicewise cross-correlation was also calculated. The DWI time-series were resampled to ACPC, generating a pre-processed DWI run in ACPC space with 2mm isotropic voxels.

##### Reconstruction

After the initial data processing, connectomes were uniformly constructed for each dataset following the workflow described in QSIPrep (Cieslak et al., 2021), and Mrtrix3 (J.-D. Tournier et al., 2019). To create the hybrid surface/volume segmentation freesurfer output and the QSIPrep pre-processed T1w images and brain masks were used (R. Smith, Skoch, Bajada, Caspers, & Connelly, 2020). Multi-tissue fibre response functions were determined using the dhollander algorithm and group average response functions were calculated. After this, the Fiber Orientation Distributions (FODs) were calculated for the gray matter, white matter and cerebrospinal fluid, with a maximum spherical harmonic degree of 8 (Lmax) (Cieslak et al., 2021), utilizind the multi-shell constrained spherical deconvolution (CSD) algorithm (dwi2fod) (J.-D. Tournier, Calamante, Gadian, & Connelly, 2004; J.-D. Tournier et al., 2008) and employing an unsupervised multi-shell multi-tissue method (Dhollander, Mito, Raffelt, & Connelly, 2019; Dhollander, Raffelt, & Connelly, 2016).

After this, mtnormalize was used to intensity-normalize the FODs (Raffelt et al., 2017). Further, 10 million probabilistic streamlines were generated using the second-order integration of the Fiber Orientation Distributions algorithm (iFOD2) (J. D. Tournier, Calamante, & Connelly, 2010), in conjugation with anatomically-constrained tractography (ACT) (R. E. Smith, Tournier, Calamante, & Connelly, 2012). Dynamic seeding (Robert E Smith, Jacques-Donald Tournier, Fernando Calamante, & Alan Connelly, 2015) was applied, with a power of 0.33 (Cieslak et al., 2021), FOD amplitude cutoff of 0.06, and a range of track lengths spanned from 5 to 300 mm, along with a max attempt per seed of 1000 (Civier, Smith, Yeh, Connelly, & Calamante, 2019). The system allowed for backtracking to adjust track lengths if unsatisfactory structural terminations were encountered (R. E. Smith et al., 2012). To ensure the biological relevance of connectivity quantifications (Robert E. Smith, Jacques-Donald Tournier, Fernando Calamante, & Alan Connelly, 2015), spherical-deconvolution informed filtering of tractograms (tckSIFT2) (Robert E Smith et al., 2015) was employed to assign a weight to each streamline. In this process, the SIFT proportionality coefficient (mu) was extracted.

Individual’s 84 x 84 connectivity matrix were generated using 84 anatomic regions of interest as nodes. The parcellation process was performed using Freesurfer (Desikan et al., 2006). The connection strength between nodes (i and j) was computed by summing the weights of the streamlines that connect them, this value was then stored in element (i,j). Before conducting any analysis, inter-nodal connections were removed (Rubinov & Sporns, 2010) and bidirectional matrices were created. For a visual summary of the primary steps involved in generating the connectivity matrices, see Figure 1.

#### Supplementary material Section D

| Table S4: Data-driven Dietary Pattern adherence groups in Each Clinical Trial – NBS and TFNBS analysis | | | | | | | | | | | | | |
| --- | --- | --- | --- | --- | --- | --- | --- | --- | --- | --- | --- | --- | --- |
|  |  |  | MeDi Tertiles (Martinez-Gonzalez et al. (2012) | | |  | DASH Quintiles (Folsom et al. (2007) | | |  | MIND tertiles (Morris et al. (2015) | | |
| **Data-Driven** | | | | | | | | | | | | | |
| Clinical trial |  |  | T1 | T2 | T3 |  | T1 | T2 | T3 |  | T1 | T2 | T3 |
| MAST | Range |  | 2.0-4.0 | 5.0-6.0 | 7.0-9.0 |  | 1.5–4.0 | 4.5–5.0 | 5.5–7.0 |  | 3.0-5.5 | 6.0–7.5 | 8.0 -11.5 |
|  | n |  | 20 | 21 | 11 |  | 20 | 14 | 18 |  | 17 | 19 | 16 |
| PLICAR | Range |  | 1.0-3.0 | 4.0-5.0 | 6.0-6.0 |  | 0.0–3.5 | 4.0-5.0 | 5.5-7.5 |  | 5.0-6.5 | 7.0-8.0 | 8.5-10 |
|  | n |  | 29 | 19 | 4 |  | 31 | 15 | 6 |  | 13 | 24 | 15 |
| *Note.* Ranges and participant distribution are provided for each adherence group within the respective clinical trials. Data-Driven adherence values for tertiles are determined through data-driven analysis. Abbreviations: n = number of participants, Q = Quintile, T = Tertiles. | | | | | | | | | | | | | |

References:

Andersson, J. L., Graham, M. S., Zsoldos, E., & Sotiropoulos, S. N. (2016). Incorporating outlier detection and replacement into a non-parametric framework for movement and distortion correction of diffusion MR images. *NeuroImage, 141*, 556-572.

Andersson, J. L., Skare, S., & Ashburner, J. (2003). How to correct susceptibility distortions in spin-echo echo-planar images: application to diffusion tensor imaging. *NeuroImage, 20*(2), 870-888.

Andersson, J. L., & Sotiropoulos, S. N. (2016). An integrated approach to correction for off-resonance effects and subject movement in diffusion MR imaging. *NeuroImage, 125*, 1063-1078.

Avants, B. B., Epstein, C. L., Grossman, M., & Gee, J. C. (2008). Symmetric diffeomorphic image registration with cross-correlation: evaluating automated labeling of elderly and neurodegenerative brain. *Medical Image Analysis, 12*(1), 26-41.

Cieslak, M., Cook, P. A., He, X., Yeh, F. C., Dhollander, T., Adebimpe, A., . . . Satterthwaite, T. D. (2021). QSIPrep: an integrative platform for preprocessing and reconstructing diffusion MRI data. *Nat Methods, 18*(7), 775-778. doi:10.1038/s41592-021-01185-5

Civier, O., Smith, R. E., Yeh, C.-H., Connelly, A., & Calamante, F. (2019). Is removal of weak connections necessary for graph-theoretical analysis of dense weighted structural connectomes from diffusion MRI? *NeuroImage, 194*, 68-81.

Dale, A. M., Fischl, B., & Sereno, M. I. (1999). Cortical surface-based analysis: I. Segmentation and surface reconstruction. *NeuroImage, 9*(2), 179-194.

Desikan, R. S., Ségonne, F., Fischl, B., Quinn, B. T., Dickerson, B. C., Blacker, D., . . . Killiany, R. J. (2006). An automated labeling system for subdividing the human cerebral cortex on MRI scans into gyral based regions of interest. *NeuroImage, 31*(3), 968-980. doi:<https://doi.org/10.1016/j.neuroimage.2006.01.021>

Dhollander, T., Mito, R., Raffelt, D., & Connelly, A. (2019). *Improved white matter response function estimation for 3-tissue constrained spherical deconvolution.* Paper presented at the Proc. Intl. Soc. Mag. Reson. Med.

Dhollander, T., Raffelt, D., & Connelly, A. (2016). *Unsupervised 3-tissue response function estimation from single-shell or multi-shell diffusion MR data without a co-registered T1 image.* Paper presented at the ISMRM workshop on breaking the barriers of diffusion MRI.

Fonov, V. S., Evans, A. C., McKinstry, R. C., Almli, C. R., & Collins, D. (2009). Unbiased nonlinear average age-appropriate brain templates from birth to adulthood. *NeuroImage*(47), S102.

Kellner, E., Dhital, B., Kiselev, V. G., & Reisert, M. (2016). Gibbs‐ringing artifact removal based on local subvoxel‐shifts. *Magnetic Resonance in Medicine, 76*(5), 1574-1581.

Klein, A., Ghosh, S. S., Bao, F. S., Giard, J., Häme, Y., Stavsky, E., . . . Chaibub Neto, E. (2017). Mindboggling morphometry of human brains. *Plos Computational Biology, 13*(2), e1005350.

Power, J. D., Mitra, A., Laumann, T. O., Snyder, A. Z., Schlaggar, B. L., & Petersen, S. E. (2014). Methods to detect, characterize, and remove motion artifact in resting state fMRI. *NeuroImage, 84*, 320-341.

Raffelt, D., Dhollander, T., Tournier, J.-D., Tabbara, R., Smith, R. E., Pierre, E., & Connelly, A. (2017). *Bias field correction and intensity normalisation for quantitative analysis of apparent fibre density.* Paper presented at the Proc. Intl. Soc. Mag. Reson. Med.

Renton, A. I., Dao, T. T., Johnstone, T., Civier, O., Sullivan, R. P., White, D. J., . . . Amos, T. J. (2024). Neurodesk: an accessible, flexible and portable data analysis environment for reproducible neuroimaging. *Nature methods*, 1-5.

Rubinov, M., & Sporns, O. (2010). Complex network measures of brain connectivity: Uses and interpretations. *NeuroImage, 52*(3), 1059-1069. doi:<https://doi.org/10.1016/j.neuroimage.2009.10.003>

Smith, R., Skoch, A., Bajada, C. J., Caspers, S., & Connelly, A. (2020). Hybrid surface-volume segmentation for improved anatomically-constrained tractography.

Smith, R. E., Tournier, J.-D., Calamante, F., & Connelly, A. (2012). Anatomically-constrained tractography: improved diffusion MRI streamlines tractography through effective use of anatomical information. *NeuroImage, 62*(3), 1924-1938.

Smith, R. E., Tournier, J.-D., Calamante, F., & Connelly, A. (2015). The effects of SIFT on the reproducibility and biological accuracy of the structural connectome. *NeuroImage, 104*, 253-265. doi:<https://doi.org/10.1016/j.neuroimage.2014.10.004>

Smith, R. E., Tournier, J.-D., Calamante, F., & Connelly, A. (2015). SIFT2: Enabling dense quantitative assessment of brain white matter connectivity using streamlines tractography. *NeuroImage, 119*, 338-351.

Tournier, J.-D., Calamante, F., Gadian, D. G., & Connelly, A. (2004). Direct estimation of the fiber orientation density function from diffusion-weighted MRI data using spherical deconvolution. *NeuroImage, 23*(3), 1176-1185.

Tournier, J.-D., Smith, R., Raffelt, D., Tabbara, R., Dhollander, T., Pietsch, M., . . . Connelly, A. (2019). MRtrix3: A fast, flexible and open software framework for medical image processing and visualisation. *NeuroImage, 202*, 116137.

Tournier, J.-D., Yeh, C.-H., Calamante, F., Cho, K.-H., Connelly, A., & Lin, C.-P. (2008). Resolving crossing fibres using constrained spherical deconvolution: validation using diffusion-weighted imaging phantom data. *NeuroImage, 42*(2), 617-625.

Tournier, J. D., Calamante, F., & Connelly, A. (2010). *Improved probabilistic streamlines tractography by 2nd order integration over fibre orientation distributions.* Paper presented at the Proceedings of the international society for magnetic resonance in medicine.

Tustison, N. J., Avants, B. B., Cook, P. A., Zheng, Y., Egan, A., Yushkevich, P. A., & Gee, J. C. (2010). N4ITK: improved N3 bias correction. *Ieee Transactions on Medical Imaging, 29*(6), 1310-1320.

Zhang, Y., Brady, M., & Smith, S. (2001). Segmentation of brain MR images through a hidden Markov random field model and the expectation-maximization algorithm. *IEEE Trans Med Imaging, 20*(1), 45-57. doi:10.1109/42.906424
